# The Adaptive Evolution of *Leuciscus waleckii* in Lake Dali Nur and Convergent Evolution of Cypriniformes Fishes Inhabiting Extremely Alkaline Environments

**DOI:** 10.1093/gbe/evad082

**Published:** 2023-05-17

**Authors:** Zhixiong Zhou, Junyi Yang, Hongzao Lv, Tao Zhou, Ji Zhao, Huaqiang Bai, Fei Pu, Peng Xu

**Affiliations:** State Key Laboratory of Marine Environmental Science, College of Ocean and Earth Sciences, Xiamen University, Xiamen, China; Fujian Key Laboratory of Genetics and Breeding of Marine Organisms, College of Ocean and Earth Sciences, Xiamen University, Xiamen, China; State Key Laboratory of Marine Environmental Science, College of Ocean and Earth Sciences, Xiamen University, Xiamen, China; Fujian Key Laboratory of Genetics and Breeding of Marine Organisms, College of Ocean and Earth Sciences, Xiamen University, Xiamen, China; State Key Laboratory of Marine Environmental Science, College of Ocean and Earth Sciences, Xiamen University, Xiamen, China; Fujian Key Laboratory of Genetics and Breeding of Marine Organisms, College of Ocean and Earth Sciences, Xiamen University, Xiamen, China; State Key Laboratory of Marine Environmental Science, College of Ocean and Earth Sciences, Xiamen University, Xiamen, China; Fujian Key Laboratory of Genetics and Breeding of Marine Organisms, College of Ocean and Earth Sciences, Xiamen University, Xiamen, China; State Key Laboratory of Marine Environmental Science, College of Ocean and Earth Sciences, Xiamen University, Xiamen, China; Fujian Key Laboratory of Genetics and Breeding of Marine Organisms, College of Ocean and Earth Sciences, Xiamen University, Xiamen, China; State Key Laboratory of Marine Environmental Science, College of Ocean and Earth Sciences, Xiamen University, Xiamen, China; Fujian Key Laboratory of Genetics and Breeding of Marine Organisms, College of Ocean and Earth Sciences, Xiamen University, Xiamen, China; State Key Laboratory of Marine Environmental Science, College of Ocean and Earth Sciences, Xiamen University, Xiamen, China; Fujian Key Laboratory of Genetics and Breeding of Marine Organisms, College of Ocean and Earth Sciences, Xiamen University, Xiamen, China; State Key Laboratory of Marine Environmental Science, College of Ocean and Earth Sciences, Xiamen University, Xiamen, China; Fujian Key Laboratory of Genetics and Breeding of Marine Organisms, College of Ocean and Earth Sciences, Xiamen University, Xiamen, China; Laboratory for Marine Biology and Biotechnology, Pilot National Laboratory for Marine Science and Technology, Qingdao, China

**Keywords:** alkaline adaptation, carbonic anhydrase, RH glycoproteins, *Leuciscus waleckii*

## Abstract

*Leuciscus waleckii* is widely distributed in Northeast Asia and has high economic value. The population in Lake Dali Nur can adapt to extremely alkaline–saline water with bicarbonate over 50 mmol/L (pH 9.6), thus providing an exceptional model for exploring the mechanisms of adaptive evolution under extreme alkaline environments. Here, we assembled a high-quality chromosome-level reference genome for *L. waleckii* from Lake Dali Nur. Based on the resequencing of 85 individuals from divergent populations, the historical population size of *L. waleckii* in Lake Dali Nur dramatically expanded in a thousand years approximately 13,000 years ago and experienced a cliff recession in the process of adapting to the alkaline environment of Lake Dali Nur approximately 6,000 years ago. Genome scans between freshwater and alkaline populations further revealed the significant selective sweep regions from Lake Dali Nur, which harbor a set of candidate genes involved in hypoxia tolerance, ion transport, acid–base regulation, and nitrogen metabolism. 5 alkali population–specific nonsynonymous mutations were identified in CA15 gene copies. In addition, two sites with convergent amino acid mutation were detected in the RHCG-a gene among several alkali environment–adapted Cypriniformes fish. Our findings provide comprehensive insight into the genomic mechanisms of *L. waleckii* and reveal their adaptative evolution under extreme alkaline environments.

**Significance**

In alkaline lakes, many Cypriniformes fish have independently evolved the ability to survive in extreme alkaline environments. *Leuciscus waleckii* in Lake Dali Nur is their typical representative. In our study, strong selective sweep signals were detected in the *L. waleckii* population of Lake Dali Nur, with similar convergent mutation sites on *rhcga* among several alkali environment–adapted Cypriniformes fish. This is the first study to investigate the genetic mechanism of adaptive evolution to an extreme alkaline environment of *L. waleckii* at a mutation-site level and more importantly highlights the importance of inspecting both carbonic anhydrase regulation and ammonia discharging mechanism in understanding the adaptive evolution of Cypriniformes fish in alkaline lakes.

## Introduction

Diverse extreme environments, including deserts, plateaus, deep seas, and saline–alkali lakes are important components of the global ecosystem. In the process of survival under these extreme environments, species generally undergo unique adaptive evolution, which is a popular research topic in the field of biological evolution (Hoffmann and Parsons 1993). Many studies have shown that the selection pressure caused by extreme environments may change the protein coding, expression pattern, copy number, or molecular function of several core genes in adaptive species (Li et al. 2013; Gaither et al. 2018; Chen et al. 2019). Moreover, different species that adapt to the same extreme environment may adopt convergent genetic evolution strategies (Chen et al. 1997). These cases of classic adaptive evolution could help us understand the origin and evolution of species and provide new avenues for reasonably developing genetic resources for rare extreme environmental adaptive species.

Alkalization and salinization, which are critical threats to inland lakes and freshwater fishery resources, are currently widespread and occur at an unprecedented rate under intensifying global warming (Kaushal et al. 2018). Usually, the pH of these alkaline–saline lakes is higher than 9.0, and the salinities may approach approximately 50% of seawater (Wilkie and Wood 1996). Such extremely alkaline environment could disrupt the acid–base balance, inhibit the excretion of nitrogenous waste, and disturb the osmotic pressure regulation of nonadapted fish. Although the effects of these extreme environmental factors on the reproduction and growth of freshwater fish are lethal, several fish can naturally survive in alkaline–saline lakes. Therefore, the adaptive evolutionary mechanism of these recurring alkaline-adaptive fish has long been of interest to evolutionary biologists (Xu, Li, et al. 2013; Xu et al. 2017; Tong et al. 2021). Generally, to avoid elevated blood pH due to respiratory alkalosis in alkaline–saline lakes, teleosts regulate the blood pH through reversible CO_2_ hydration/dehydration reactions catalyzed by carbonic anhydrases (CAs) (Gilmour 2012). Additionally, several important ion transport channels are also involved in the regulation of acid–base balance, such as Cl^−^/HCO_3_^−^ and Na^+^/H^+^ exchangers across the gill, which has been confirmed in Lahontan Cutthroat trout (*Oncorhynchus clarkii henshawi*), rainbow trout (*Oncorhynchus mykiss*), and naked carp (*Gymnocypris przewalskii*) (Galat et al. 1985; Goss et al. 1992; Zhang et al. 2015). To deal with the inhibition of ammonia excretion under highly alkaline environment, freshwater teleosts have a variety of coping strategies, such as reducing the metabolic rate, actively excreting ammonia in the gill, converting accumulated nitrogenous waste to nontoxic glutamine or free amino acids, and synthesizing urea (Randall et al. 1989; Iwata et al. 2000; Wang et al. 2003; Ip and Chew 2010). The classic case is the Magadi tilapia (*Alcolapia grahami*), which inhabits Lake Magadi, with high pH (∼10) and salinity (∼60% of seawater) (Randall, et al. 1989). Transcriptome evidence showed that the Magadi tilapia had a functional ornithine–urea cycle pathway in the gills, which was conducive to increasing nitrogenous waste efficiency by excreting urea (Kavembe et al. 2015). Therefore, exploring the adaptive evolution of fish that can survive in an extremely alkaline environment can help to exploit the fishery potential in alkaline–saline lakes and provide new perspectives on the genetic mechanism of important physiological regulation in teleost fish (Wilkie and Wood 1996).

Amur Ide (*L. waleckii*) is a common freshwater fish in Northeast China with high economic value and is a food source for migrating birds from Siberia (supplementary fig. S1, Supplementary Material online) (Zhang et al. 2008). As an extreme example, a special Amur Ide population can survive in the extreme alkaline environment of Lake Dali Nur located on the eastern Inner Mongolia Plateau with an average altitude of 1,226 m above sea level, which is a typical saline–alkaline lake with high concentrations of carbonate salts that is lethal to most freshwater teleosts. Affected by the monsoon, Lake Dali Nur began to shrink rapidly approximately 6,600 years ago. Because Lake Dali Nur is located inland in Northeast Asia, the warm, humid summer monsoon in East Asia cannot bring precipitation to it. In winter, Lake Dali Nur was controlled by the dryly cold monsoon from the Siberian and Mongolian Plateau (Xiao et al. 2008). Due to the continuous evaporation of water caused by the dry monsoon, the area of Lake Dali Nur has decreased sharply, and water began to alkalize. Currently, the pH value of Lake Dali Nur ranges from 8.25 to 9.6, with an alkaline content (ALK) over 50 mg/L and a salinity of approximately 6‰ (Xiao, et al. 2008). Besides, Lake Dali Nur can experience a freezing period of approximately 5 months every year (Ma et al. 2019). Therefore, enduring long-term hypoxia is also key to the survival of fish in Lake Dali Nur. Based on geological and biological evidence, the prevailing view is that the Amur Ide population in Lake Dali Nur was a freshwater fish that evolved quickly in the past several thousand years and has developed great tolerance to high alkalinity (Geng and Zhang 1988). It is a model species used to explore the adaptation of teleosts to extreme alkaline environments because it has different populations living in alkaline and freshwater areas (Xu, Li, et al. 2013). Hence, scientists have been interested in the mechanism of its microevolution in the past decade, as the species rapidly evolved to survive rapid paleoenvironmental changes since the early Holocene (Wang et al. 2013; Xu, Ji, et al. 2013; Xu et al. 2017; Wang et al. 2021; Zhao et al. 2021). However, the genomic signature and key adaptive evolutionary loci underlying the tolerance to high-alkali conditions should be further explored, particularly through comparative genetic analysis between alkaline and freshwater Amur Ide populations.

Here, we present a high-quality chromosome-level genome of *L. waleckii* inhabiting the extremely alkaline waters of Lake Dali Nur. Comparative genomics analysis between *L. waleckii* and related species revealed a series of adaptive evolution events in the *L. waleckii* genome in response to an extremely alkaline environment, with regard to transposable elements and selection pressure. Based on the resequencing data from alkaline and freshwater *L. waleckii* populations, population analysis revealed the historical population size fluctuations of Lake Dali Nur *L. waleckii*. Microevolution scanning and different gene expression analysis between alkaline and freshwater populations explained the physiological regulatory mechanism and revealed candidate selected genes in the Lake Dali Nur Amur Ide population. Finally, the in-depth analysis of the CA and RH glycoprotein gene family revealed that they played important roles in the adaptation of the Lake Dali Nur *L. waleckii* to an extremely alkaline environment.

## Results

### Genome Assembly and Annotation

A high-quality chromosome-level genome is needed for the downstream analysis of adaptive microevolution. Based on the method described in Zhou et al. (2019), the genome size was evaluated to be approximately 1,125.03 Mb and the heterozygous rate and repeat rate were evaluated as 0.56% and 57.61%, respectively, by 17-mer analysis (supplementary fig. S2 and table S2, Supplementary Material online) (Zhou et al. 2019). Using the PacBio platform, we sequenced the genome of Amur Ide in Lake Dali Nur. The assembled genome spanned 1,103 Mb, with a contig N50 length of 1.52 Mb (supplementary table S4, Supplementary Material online). Genome annotation showed that the Amur Ide genome comprises approximately 49.92% repetitive sequences (supplementary tables S5 and table S6, Supplementary Material online), which was comparable to the repeat content of other Cypriniform species genomes (Wang et al. 2015; Xu et al. 2019; Chen et al. 2021). In the Amur Ide assembly, we predicted 27,633 protein-coding genes, of which 96.3% of the protein sequences showed similarity to protein sequences in public databases (supplementary tables S7 and S8 and figs. S3 and S4, Supplementary Material online). The contigs were then anchored and oriented into a chromosomal-scale assembly using the Hi-C scaffolding approach. Ultimately, we obtained a draft genome assembly of 1,105 Mb in length, with a scaffold N50 value of 39.64 Mb (supplementary table S9, Supplementary Material online). The genome assembly contained 25 chromosomes, with chromosome lengths ranging from 28.42 to 71.37 Mb and covered 1,020 Mb (92.32%) of the *L. waleckii* assembly (supplementary table S9 and figs. S5 and S6*A*, Supplementary Material online). BUSCO analysis showed that the assembly retrieved 96.4% of the conserved single-copy orthologous genes (supplementary table S10, Supplementary Material online). In addition, we mapped Illumina short reads to the *L. waleckii* reference genome with a mapping ratio of 99.05% and generated 2,707,134 SNPs (supplementary tables S11 and S12, Supplementary Material online). This evidence supported the high-quality assembly of the *L. waleckii* genome.

### Comparative Genomics between *L. waleckii* and the Related Species

**Fig. 1.**
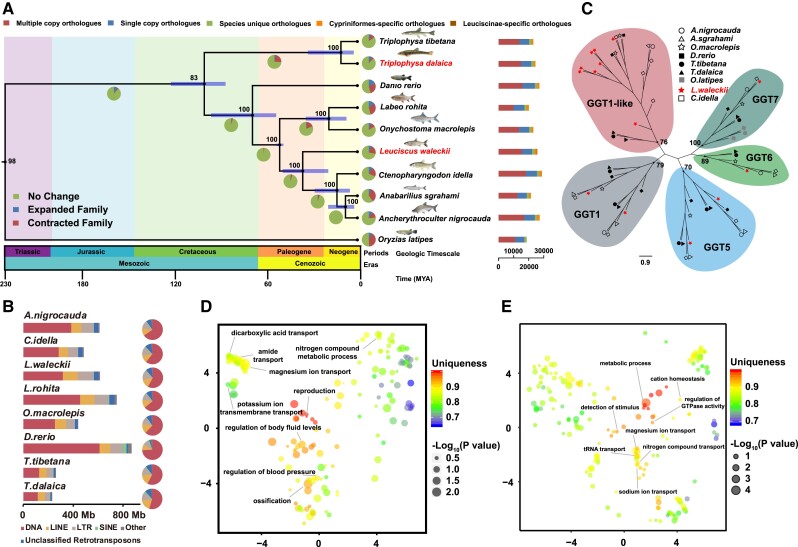
Comparison of evolutionary features of *L. waleckii* and other Cyprinidae species. (*A*) Divergence times and distribution of different types of orthologues in representative species. The blue bars in the ancestral nodes indicate the 95% confidence intervals of the estimated divergence time (Ma, million years ago). The bootstrap values of each node are displayed. (*B*) Comparison of various transposable elements between *L. waleckii* and related species. (*C*) Phylogenetic tree of GGT proteins in vertebrates showing gene expansion in the *L. waleckii* genome. The bootstrap values of the node of each gene cluster are displayed. (*D*) GO terms of positively selected genes that are summarized and visualized as a REVIGO scatter plot; (*E*) GO terms of rapidly evolving genes that are summarized and visualized as a REVIGO scatter plot. Each circle represents a cluster of related GO terms, with a single term chosen by REVIGO as the cluster representative. Clusters are plotted according to semantic similarities to other GO terms (adjoining circles are most closely related). “Uniqueness” (the negative of average similarity of a term to all other terms) measures the degree to which the term is an outlier when compared semantically with the whole list.

To determine the phylogenetic relationship of *L. waleckii* in Cypriniformes species, we compared ten teleost genomes. The phylogenetic tree revealed that *L. waleckii* and *Ctenopharyngodon idella* separated approximately 36.2 Ma (confidence interval 19.3 to 49.5 Ma), which corroborates the results of other recent studies based on smaller data sets (fig. 1*A* and supplementary table S13, Supplementary Material online) (Xu et al. 2019; Chen et al. 2021). The chromosome synteny comparisons among *L. waleckii*, *Ancherythroculter nigrocauda*, and *C. idella* showed that chromosomes 10 and 22 of *L. waleckii* fused into one chromosome in *A. nigrocauda* and *C. idella* (supplementary figs. S6*B* and S7, Supplementary Material online). The adaptive evolution of transposable elements (TEs) and gene regulation of the insertion region of TEs may have important evolutionary effects in the process of species adapting to extreme environments (Schrader and Schmitz 2019). Hence, we used a common protocol to identify TE in *L. waleckii* and seven Cypriniformes species to compare the TE contents, types, and divergence (see Methods). In the *L. waleckii* genome, approximately 598 Mb (54.17% of the genome size) were composed of TEs (supplementary table S14, Supplementary Material online). Among the interspersed repeats, the most abundant transposable elements were DNA transposons (28.59% in genome). Retrotransposons were the second most abundant repeat elements (22.85% in genome), including three major families of long terminal repeats (LTRs), long interspersed elements (LINEs), and short interspersed elements (SINEs). The TE contents and their proportions in *L. waleckii* were similar to those in other Cyprinidae fish genomes except for the LTRs and LINEs, in which LTRs were significantly expanded to 11.47% in *L. waleckii*, and LINEs were significantly expanded to 10.87% in *L. waleckii* (fig. 1*B* and supplementary table S14, Supplementary Material online). The insertion time of TEs can be estimated based on their Kimura substitution level. Compared with those in *A. nigrocauda* and *C. idella*, we found that the LTRs and LINEs in *L. waleckii* significantly expanded with 15% divergence rates (supplementary fig. S8, Supplementary Material online). In another extremely alkaline environment–adapted Cyprinidae fish, *Triplophysa dalaica*, the LTRs and LINEs also expanded with 13% divergence rates compared with those of *Triplophysa tibetana*, which inhabits a freshwater environment. Besides, the burst of LTR and LINE in *L. waleckii* is concentrated at *K* < 8, compared with *C. Idella*, and the burst of LTR and LINE in *T. dalaica* is concentrated at *K* < 14, compared with *T. tibetana* (supplementary fig. S9, Supplementary Material online). Hence, the expansion of LTRs and LINEs in two Lake Dali Nur–specific fish may contribute to their adaptation to an extremely alkaline environment.

### Expanded Gene Families Underlying Alkaline Adaptation of *L. waleckii*

The expansion and contraction of gene families may play a key role in adaptation and tolerance to extreme environments (Demuth and Hahn 2009). We uncovered 1,751 *L. waleckii* gene families with expansion and 5,202 families with contraction (fig. 1*A* and supplementary table S15, Supplementary Material online). Gene Ontology (GO) enrichment analysis showed that the expanded gene families were mainly involved in cell death, lipid transport, glutathione catabolic processes, and chromatin assembly, and the contracted gene families were mainly involved in ion transport, germ cell development, calcium ion homeostasis, and cell recognition (supplementary tables S16 and S17 and figs. S10 and S11, Supplementary Material online). We identified 14 copies of gamma-glutamyl transferase (GGT) genes in *L. waleckii*, which were considerably expanded compared with those in other Cyprinidae species (fig. 1*C* and supplementary table S18, Supplementary Material online). Of these GGT genes, GGT1-like genes were expanded to eight copies in *L. waleckii*, which may enhance its synthetic capacity of less toxic glutamine and glutathione.

### Positively Selected and Rapidly Evolving Genes in *L. waleckii*

In the process of adapting to extreme environments, natural selection will form selection marks on several important genes, among which the most important types are positive selection genes (PSGs) and rapid evolution genes (REGs) (Chen et al. 2019). PSGs were the genes that were positively selected during the evolution of the species (dN/dS > 1) (Kryazhimskiy and Plotkin 2008). REGs were the genes with larger dN/dS values in *L. waleckii* than other species. Finally, we identified a set of 131 REGs in the *L. waleckii* lineage, including glutathione peroxidase 7 (*gpx7*), AMP deaminase 2 (*ampd2*), and others (supplementary tables S19 and S20, Supplementary Material online). In addition, we identified 369 PSGs in *L. waleckii*, including uromodulin-like 1 (*umodl1*), ammonium transporter Rh type A (*rhag*), glutamate receptor ionotropic, delta-1 (*grid1*), and others (supplementary tables S21 and table S22, Supplementary material online). By applying the GO clustering tool REVIGO (Supek et al. 2011) to the terms associated with the REGs from *L. waleckii*, we found that terms related to reproduction, ossification, and blood circulation had low average similarity (i.e., higher unique; uniqueness > 0.9) (fig. 1*D* and supplementary table S23, Supplementary Material online). In addition, several terms related to ion transport, energy metabolism, and ammonia nitrogen metabolism had medium average similarity (medium unique; 0.7 < uniqueness < 0.9), which suggests that they were important in the adaptive evolution of extreme alkaline environments (Xu et al. 2017). Conversely, in PSGs, terms related to ion transport, metabolic process, and ammonia nitrogen metabolism had low average similarity (i.e., higher unique; uniqueness > 0.9), which indicates that a higher proportion of REGs in the *L. waleckii* genome may be involved in the adaptive evolution to an extreme alkaline environment (fig. 1*E* and supplementary table S24, Supplementary Material online).

### Differential Gene Expression under Alkaline Stress

Previously, several differential gene expression analyses based on RNA-Seq data had detected lots of differentially expressed genes (DEGs) between ALK and FW populations. To provide gene expression level evidence linking the REGs, PSGs, and expanded genes, we collected fresh gill kidney and liver tissue from ALK and FW population and analyzed the RNA-seq data based on the new chromosome-level *L. waleckii* genome (supplementary table S25, Supplementary Material online). The results demonstrated 5,014, 4,848, and 4,468 DEGs in the gill kidney and liver, respectively (supplementary tables S26 and S34 and fig. S12, Supplementary Material online). There were 280 and 232 GO terms were identified from upregulated and downregulated DEGs in the gill, which the top ones were metabolic process (GO: 0008152) and GTP binding (GO: 0005525), respectively (supplementary tables S35 and S36, Supplementary Material online). There were 167 and 47 GO terms identified from upregulated and downregulated DEGs in the gill, which the top ones were catalytic activity (GO:0003824) and chromosome (GO: 0005694), respectively (supplementary tables S37 and S38, Supplementary Material online). There were 89 and 14 GO terms identified from upregulated and downregulated DEGs in the gill, which the top ones were intracellular (GO: 0005622) and extracellular region parts (GO: 0044421), respectively (supplementary tables S39 and S40, Supplementary Material online). 170 PSGs and 69 REGs were identified with differential expression (supplementary tables S41 and S42, Supplementary Material online). For example, we found *rhag*, *ampd2*, and *umodl1* were downregulated expressions in the kidney, which is related to ammonia nitrogen metabolism. In the glutamate metabolic pathway, *gpx7* showed significantly decreased expression in the liver and *grid1* also showed decreased expression in the gill in ALK samples. Besides, we found at least two GGT1 genes, two GGT1-like genes, two GGT5 genes, and one GGT6 gene which were differently expressed in ALK samples, which were identified as belonging to expanded gene families (supplementary table S37, Supplementary Material online). The results provided gene expression–level evidence that these PSGs and REGs might be associated with extreme alkaline stress and adaptive evolution.

### Population Genomic Analysis of *L. waleckii*

**Fig. 2.**
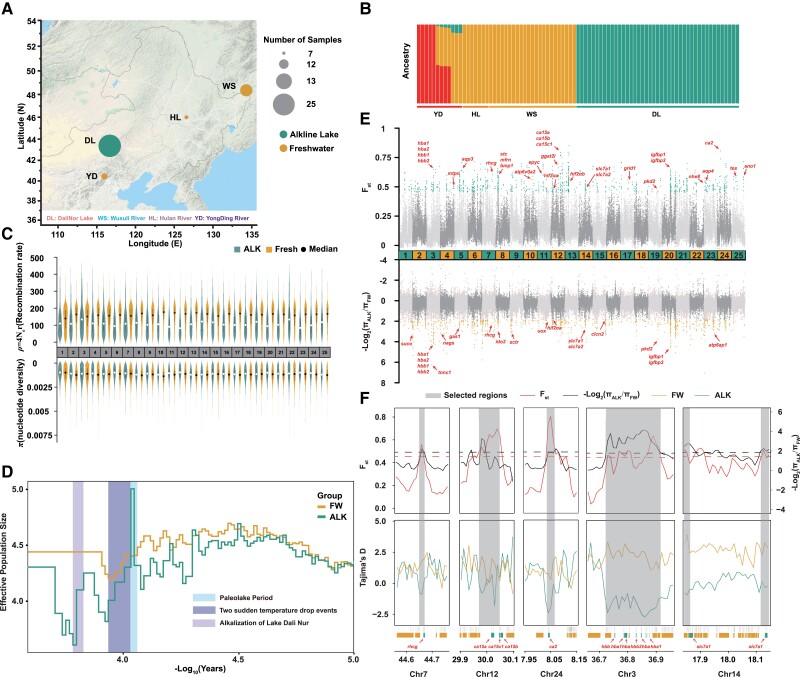
The population genetic of the *L. waleckii* populations and genomic regions of the selected ALK population. (*A*) Geographic locations of sample collection. (*B*) The structure analysis of 85 *L. waleckii* (*K* = 3) samples. (*C*) The recombination and nucleotide diversity statistics for 25 chromosomes between the ALK and FW populations. (*D*) The effective population size and demographic history of the ALK and FW populations of *L. waleckii*. (*E*) Selective scanning of the *F*_st_ and *π* ratio values between the ALK and FW *L. waleckii* populations. The important candidate genes are identified by red arrows. (*F*) Selective scanning of 13 selected genes. The *F*_st_, *π* ratios, and Tajima's *D* values are plotted using 10,000 bp sliding windows. Genome annotations are shown at the bottom.

*L. waleckii* was widely distributed in the aquatic ecosystem of Northeast China. Due to the extremely alkaline environment of Lake Dali Nur, the *L. waleckii* population is a marvelous example of rapid adaptive evolution. We collected 25 *L. waleckii* individuals from the alkaline environment of Lake Dali Nur (DL) and 32 *L. waleckii* individuals from several freshwater locations (Wusuli river [WS], Hulan river [HL], and Yongding river [YD]), respectively. Combining these individuals with previous resequencing data, which contained 18 DL individuals and 10 WS individuals, we could compare the alkaline population with freshwater populations to investigate their genetic variation and the selective signatures of adaptive evolution (Xu et al. 2017) (fig. 2*A* and supplementary table S43, Supplementary Material online). After removing the low-quality sites, we identified a total of 6,206,224 SNPs and 845,427 insertions/deletions (INDELs) (supplementary table S44, Supplementary Material online). Based on a phylogenetic tree, 85 individuals were grouped into three distinct clades, including the WS–HL group, DL group, and YD group (supplementary fig. S12, Supplementary Material online). Similar results were obtained by PCA and admixture analysis, with the DL and WS–HL populations being completely differentiated (fig. 2*B*and supplementary fig. S13, Supplementary Material online). Compared with the DL and WS–HL populations, three YD individuals had a large proportion of genetic components from the WS–HL population mixed with a small proportion of genetic components from the DL population. These results suggested that some individuals from the WS–HL population inhabit the YD river and may have exchanged genes with the DL population. In addition, four YD individuals contained equivalent genetic components from the YD and WS–HL populations and showed some admixture with a small proportion of genetic components from the DL population. Hence, we chose only the WS–HL population to represent the freshwater population. Because seven YD populations showed different degrees of genetic infiltration from DL populations, the remaining five purebred YD individuals had no statistical significance due to the small number of individuals. Natural selection should leave several clear population genetic signatures based on survival in the extremely alkaline environment. Using the DL population as the alkaline-surviving population (ALK) and WS–HL as the freshwater-inhabiting population (FW), we found that the *π* values of the ALK population was significantly lower than that of the FW population across the 25 chromosomes (*t*-test, *P* = 1.1 × 10^−4^) (fig. 2*C* and supplementary table S45, Supplementary Material online). Statistical analysis also showed that the recombination rate of ALK was significantly lower than that of the FW population (fig. 2*C* and supplementary table S45, Supplementary Material online). These results supported the hypothesis that *L. waleckii* may experience an intense selective sweep in the process of adapting to an extreme alkaline environment.

### Demographic and Geographic History

We reconstructed the demographic history of the ALK and FW populations. The results showed that approximately 13,000 years ago, the ALK population size dramatically expanded over a thousand years, this is consistent with the rapid expansion of Lake Dali Nur during this period (fig. 2*D* and supplementary fig. S14, Supplementary Material online) (Lan et al. 2018). After that, the ALK population experienced two significant sharp population declines at approximately 8,400 and 6,600 years ago, which reached its smallest population size at about 6,000 years ago (fig. 2*D*). In this process, the ALK population gradually adapted to the extreme alkaline environment in Lake Dali Nur. Differently, the FW population only experienced one significant sharp population declines at about 9,000 years ago. Undoubtedly, two bottleneck effects experienced by the ALK population in the process of adapting to an alkaline environment eventually led to its lower recombination rate and nucleotide diversity compared with the FW population.

### Selective Signatures Underlying Alkaline Adaptation in the *L. waleckii* Population

Natural selection could leave imprints on specific regions in the genome, such as highly differentiated genetic loci and significant changes in genetic diversity. To identify the candidate genomic regions under selective sweeps in the ALK genome, we scanned the genome-wide variations and allele frequency spectra based on approximately 7.0 million SNPs and INDELs. We identified 494 and 488 candidate genes by *F*_st_ and *π* ratios (*π*_FW/ALK_), respectively; these genes were related to hypoxia tolerance, ion transport, acid–base regulation, and nitrogen metabolism (fig. 2*E* and supplementary tables S46–S49, Supplementary Material online). The enrichment categories of 788 candidate genes were associated with oxygen transport (GO:0015671), metal ion binding (GO:0046872), transmembrane transport (GO:0055085), carbonate dehydratase activity (GO:0004089), nitrogen compound transport (GO:0071705), and amino acid transport (GO:0006865), possibly suggesting selection pressure on hypoxia tolerance, ion transport, acid–base regulation, and nitrogen metabolism during adaptation to the extreme environment in Lake Dali Nur (supplementary fig. S15 and tables S50 and S51, Supplementary Material online). Combined with DEGs, 99 candidate selected genes were identified with differential expression (supplementary fig. S16, Supplementary Material online).

### Adaptive Evolution of CA in the Lake Dali Nur Population

**Fig. 3.**
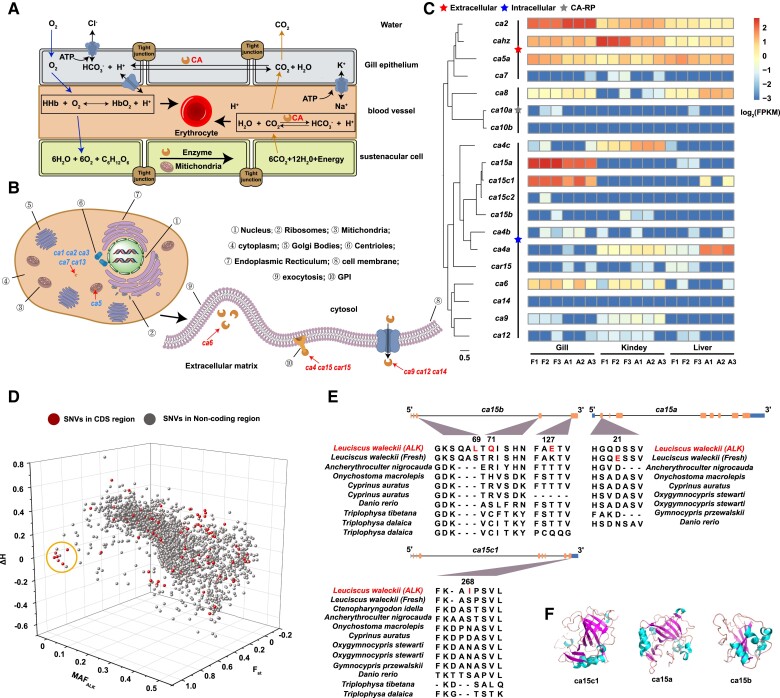
Adaptive evolution of the CA gene family in *L. waleckii.* (*A*) The regulatory mechanism of acid–base balance in Cyprinidae fish gills. (*B*) The distribution of three CA groups according to subcellular localization and catalytic activity. (*C*) The phylogenetic tree of 19 CA genes in *L. waleckii* and the gene expression heatmap in the gill, liver, and kidney between the ALK and FW populations. (*D*) 3D plot visualizing the highly differentiated SNVs between the ALK and FW populations. The *x* axis represents the minimum allele frequency (MAF_ALK_) in the ALK population. The *y* axis represents the *F*_st_ between the ALK and FW populations. The *z* axis represents the difference in heterozygosity (Δ*H*) between the ALK and FW populations. The highly differentiated SNPs are framed with circles. (*E*) The highly differentiated nonsynonymous SNP mutation in three copies of CA15 and the protein-coding genes of species related to *L. waleckii*. The mutated amino acid is indicated in red. The CDS region is represented by the orange bar, and the UTR is represented by the blue bar. (*F*) The 3D structure of three copies of CA15 in the ALK *L. waleckii* population.

Fish living in Lake Dali Nur need to survive in a continuous carbonate alkaline environment. For most freshwater teleosts, intracellular acid–base regulation occurs by the excretion or uptake of carbon dioxide (CO_2_) and HCO_3_^−^ through the reversible hydration/dehydration reactions of CO_2_: $CO_{2}+H_{2}O↔{\mathrm{HCO}}_{3}^{\text{-}}++OH^{\text{-}}$. CAs, as the key zinc metalloenzymes, can catalyze reversible CO_2_ hydration/dehydration reactions, which is conducive to maintaining acid–base balance and homeostasis of the internal environment (fig. 3*A*) (Henry 1996; Gilmour and Perry 2009). Among vertebrates, CAs are divided into three groups according to subcellular localization and catalytic activity (fig. 3*B*) (Ferreira-Martins et al. 2016). In our genome-wide scan for signatures of selection, four CA genes were identified with significant differentiation signals between ALK and FW populations (fig. 2*E*and fig. 2*F*). Furthermore, we used 18 CAs of zebrafish as queries and identified 19 CAs in *L. waleckii* (supplementary figs. S17 and S18 and tables S52 and S53, Supplementary Material online). In *L. waleckii*, CA15c has two copies, where it only has one copy in zebrafish (supplementary table S54, Supplementary Material online). Compared with the FW population, we observed decreased expression of CA5a, CA9, CA15a, and CA15c1 and elevated expression of CA2 and CAr15 in the gills of the ALK population (fig. 3*C*). In the kidney, CAhz was downregulated, and CA2, CA4a, and CA4c were upregulated. In addition, CA2 and Car15 were downregulated, and CA4a and CA8 were upregulated in the liver. To accurately locate the adaptive evolutionary sites of CAs, we calculated the *F*_st_, heterozygosity, and allele frequency of 3,721 SNPs and INDELs in 19 CAs of *L. waleckii* (fig. 3*D* and supplementary table S54, Supplementary Material online). Twenty SNPs and two INDELs were highly differentiated (*F*_st_ > 0.9; MAF_ALK_ < 0.1) between the ALK and FW populations, including five nonsynonymous SNPs and one synonymous SNP (supplementary table S55, Supplementary Material online). Interestingly, all 22 SNVs were distributed within three CA15 copies on chromosome 12. In CA15a, a nonsynonymous SNP was identified in exon 2, which caused an amino acid change from glutamic acid (E) to aspartic acid (D) in the FW *L. waleckii* population (fig. 3*E*). In CA15c, a nonsynonymous SNP was identified in exon 9. In addition, a SNP mutation was also detected in the 3′ UTR of CA15c. In CA15b, three nonsynonymous SNPs were identified in exons 3, 4, and 5. Comparison with related species showed that these amino acid mutations existed only in the ALK *L. waleckii* population (fig. 3*E*). The reconstruction of the 3D model of CA protein from zebrafish showed that these mutations did not change the spatial structure of the three CA15 copies in alkaline and freshwater *L. waleckii* populations (supplementary fig. S19, Supplementary Material online). Nevertheless, these alkaline-specific amino acid mutations may change the catalytic activity of their encoded CA15 genes.

### Convergent Evolution of *rhcg* in Alkaline-adapted Cyprinidae Species

**Fig. 4.**
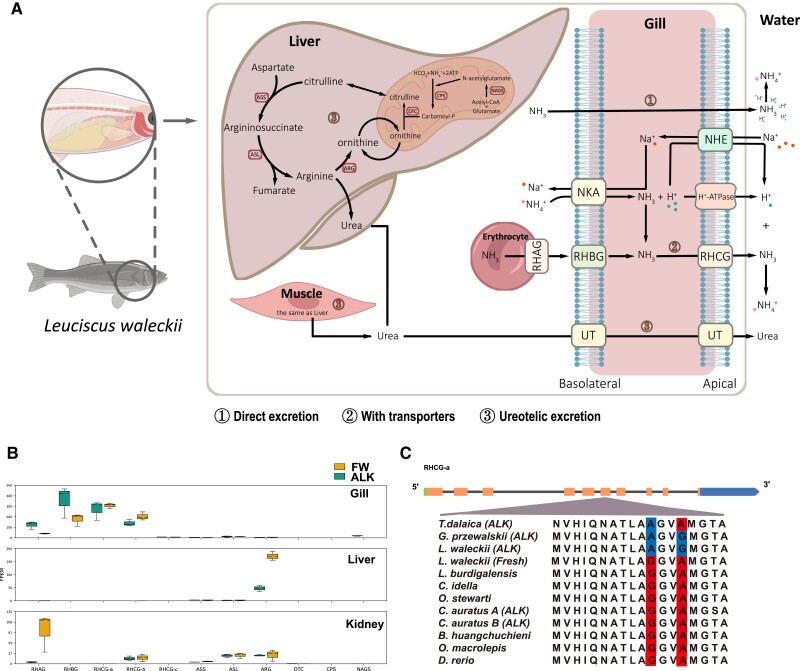
Schematic diagram of the ammonia nitrogen metabolism mechanism in *L. waleckii.* (*B*) Gene expression heatmap of RH glycoproteins in the gill, liver, and kidney between the ALK and FW populations. (*C*) The highly differentiated nonsynonymous SNP mutation in RHCG-a and the protein-coding genes of species related to *L. waleckii*. The mutated amino acid in the ALK population is indicated in red, and the amino acid in FW is indicated in blue.

Nitrogen metabolism occurs continuously in animals, resulting in the accumulation of toxic ammonia that needs to be excreted or detoxified (Ip and Chew 2010). For fish living in freshwater, ammonia can easily cross the gills, so it is usually considered to be excreted directly, mainly through Rhesus glycoproteins (Rh) (fig. 4*A*) (Ip and Chew 2010). In the *L. waleckii* ALK population, the ammonium transporter Rh type C (*rhcg-a*) was found within the selected sweep region on chromosome 7 (figs. 2*E*and 2*F*). To clarify the potential mechanism of ammonia excretion of alkali-adapted *L. waleckii* among such variable pathways, related genes that associated with excreting ammonia were identified and searched across the whole genome (Biver et al. 2008; Braun et al. 2009; Wright and Wood 2009; Ip and Chew 2010; Wood et al. 2013). Then, the abundance of the corresponding mRNAs represented the expression of each gene and helped determine which pathway was dominant. Our RNA-seq results implied that for *L. waleckii*, the Rh family, especially glycoprotein members, plays the main role in ammonia excretion in the gill (fig. 4*B*). Hence, we identified seven RH proteins in *L. waleckii* that had six shared motifs (supplementary figs. S20 and S21 and table S56, Supplementary Material online). According to the genome-wide SNP database constructed previously, a total of 764 SNVs from Rh glycoproteins were identified (supplementary table S57, Supplementary Material online). Subsequently, we found five SNPs with a higher *F*_st_ (>0.8) and located within CDS areas (supplementary table S58, Supplementary Material online). All these SNPs are nonsynonymous mutations in the *rhcga* gene. *rhcg* genes were able to move NH_3_ across the apical membrane of the branchial structure (Wright and Wood 2009).

Furthermore, in addition to Amor Ide living in such an alkaline water area, previous studies have determined that other teleost species have evolved some characteristics to survive in the same lake (*T. dalaica* and *Carassius auratus*) or other similar conditions (*G. przewalskii* in Qinghai Lake) (Luo et al. 2020; Tong et al. 2021; Zhou et al. 2021). With enough genetic information on these different species living under similar conditions, we checked all the *rhcga* alignments based on sequence similarity and discovered high convergence among four alkaline survivors at two of the seven loci that differentiated freshwater and alkali water *L. waleckii* populations. *G*. *przewalskii* shared two same amino acid substitutions in exon 6 (G-A and A-G), and *T. dalaica* shared a same amino acid substitution in exon 6 (G-A) (fig. 4*C*). The *C. auratus* in Lake Dali Nur maintained the same amino acids as other freshwater fish in these mutation sites. The 3D reconstruction of the protein model showed that the Rhcha protein 3D structure was different between ALK and FW populations, but the 3D structure of the location of two mutation sites is consistent between them (supplementary fig. S22, Supplementary Material online).

## Discussion

In this paper, comparative genomics implicated several specific characteristics of adaptive changes in *L. waleckii* regarding gene expansion, transposable elements, and selection pressures. As an important regulator in the glutamine and glutathione metabolic pathway, gamma-glutamyl transferase (GGT) cleaves the gamma-glutamyl bond, releases free glutamate and the dipeptide cysteinylglycine, and transfers the gamma-glutamyl moiety to an acceptor amino acid to form a new gamma-glutamyl compound (Balen et al. 2012). In addition, increased plasma GGT has been confirmed to accelerate the rate of ammonia synthesis in blood and plasma (da Fonseca-Wollheim 1990). There are usually six to ten copies of GGT genes in Cyprinidae species, including GGT1, GGT1-like, GGT5, GGT6, and GGT7. Of these GGT genes, GGT1-like genes were expanded to eight copies in *L. waleckii* (fig. 1*C* and supplementary table S18, Supplementary Material online). Therefore, the expansion of GGT1-like genes in the *L. waleckii* genome might be among the adaptive changes that enhance the synthetic capacity of less toxic glutamine and glutathione.

Population genomic analysis showed that ALK population had lower nucleotide diversity and recombination rate than FW population, which suggested that the ALK population may have experienced severe population decline and large-scale selective sweep. Based on the reconstruction of demographic history between ALK and FW populations, we found that the ALK population size dramatically expanded over a thousand years approximately 13,000 years ago, which Lake Dali Nur also experienced a rapid expansion during this period (fig. 2*D*). In this period, with the end of the last glacial period, an ancient Lake Dali Nur with a wide drainage area was formed by the convergence of many glacial meltwater layers (supplementary fig. S14, Supplementary Material online) (Lan et al. 2018). With two sudden temperature drop events (Younger Dryas and 8200 BP cold event), the historical populations of both ALK and FW experienced different degrees of decline (fig. 2*D*). In this period, Lake Dali Nur entered a phase of slow contraction (Lan et al. 2018). Since 6,600 years ago, due to the continuous evaporation of water caused by the dry monsoon, the area of Lake Dali Nur has decreased sharply, and water began to alkalize (Xiao et al. 2008). In this process, the ALK population size dropped and reached its lowest level approximately 6,000 years ago. Subsequently, the ALK population gradually adapted to the extreme alkaline environment. Moreover, the ALK population began to recover through opportune occupation of the vacant ecological niche that was caused by the mass extinction of other fishes in Lake Dali Nur in this period.

Combined with genome-wide select signal scan and compare transcriptome analysis, we identified several candidate genes which were associated with hypoxia tolerance, ion transport, acid–base regulation, and nitrogen metabolism during adaptation to the extreme environment in Lake Dali Nur. Especially, we identified 12 hemoglobin subunit (*hba*) and 5 hemoglobin subunit beta (*hbb*) genes on chromosome 3, that showed a significant selective sweep signal (fig. 2*E*). Among vertebrates, hemoglobin plays a pivotal role in adapting to long-term high-altitude hypoxic environments. For example, several positive selection sites were identified in the Hb genes of Schizothoracinae fishes, and they may accelerate the process of the functional divergence of Hb isoforms (Lei et al. 2021). In addition, two copies of the endothelial PAS domain–containing protein 1 (*hif2α*) gene, which encodes the transcription factor HIF2α, were detected by *F*_st_ and *π* ratio analysis (fig. 2*E*). We also observed elevated expression of *hif1α* and *hif2α* in the gills of alkaline-acclimated *L. waleckii* compared with the freshwater population (supplementary table S36, Supplementary Material online). These genes could accelerate erythrocyte synthesis and increase the concentration of hemoglobin in blood (Tian et al. 1997). Furthermore, based on RNA sequencing, *hif1αB* and *hif2αA* might be involved in the high-altitude hypoxia adaptation of *T. dalaica* (Wang et al. 2015). These evidences implied that *hb* and *hif2α*, as the key hypoxia response genes, evolved quickly to adapt to the high-altitude environment.

Due to the important role of CA genes in intracellular acid–base regulation by catalyzing reversible hydration/dehydration reactions of CO_2_, we identified 19 CAs in *L. waleckii* and detected five alkali population–specific nonsynonymous mutations in three CA15 gene copies. In the classical acid–base regulation model in zebrafish, apical H + -ATPase and sodium–hydrogen exchanger 3b (NHE3b) NHE3b provide H^+^ for the CA15-catalysed CO_2_ dehydration reaction, which generates CO_2_ that enters HRCs (Gilmour 2012; Lin et al. 2015). In contrast, HRCs express cytosolic CA2-like to catalyze CO_2_ hydration and secrete HCO_3_^−^ out of the cell by anion exchange. In Lake Dali Nur, *L. waleckii* upregulated CA15a and CA15c1 to transform excess HCO_3_^−^ to CO_2_. However, downregulation of CA2 is a plausible mechanism for maintaining the acid–base balance of the gill, via reducing the synthesis rate of HCO_3_^−^ by intracytoplasmic hydration of CO_2_. Another hypothesis supported that apical membrane–bound CA (CA15a) in the gill could catalyze the CO_2_ hydration and provide the excess protons to NH_3_ to form NH_4_^+^ (Wright and Wood 2009). Intracellularly, NH_4_^+^ releases H^+^ to form NH_3_, which is transported to the external environment by Rhcga. In these processes, the hydrogen potential difference between the intracellular and extracellular space facilitates Na^+^/H^+^ exchange. However, our expression data for the ALK *L. waleckii* population showed that *slc26a6* was upregulated in the gill, which demonstrates a role of Cl^−^/HCO_3_^−^ exchangers in Cl^−^ uptake (supplementary table S27, Supplementary Material online). Hence, it is more likely that CA15a catalyzes CO_2_ dehydration in ALK *L. waleckii* gills, which contributes to the bicarbonate potential difference between the inside and outside of the cell membrane.

To inhibit the direct emission of ammonia is another fatal threat of the extreme alkaline environment to freshwater fish, because released NH_3_ cannot be trapped by insufficient H^+^ in some aquatic environments, such as alkaline Lake Dali Nur, making it nearly impossible to sustain an ammonia concentration gradient outside the gill (Wood et al. 2013). Our RNA-seq results implied that for *L. waleckii* in Lake Dali Nur, the Rh family, especially glycoprotein members, plays the main role in ammonia excretion (fig. 4*B*). We found five nonsynonymous SNPs in ALK population, which were a homozygous genotype of rare alleles. From our results, the alkaline water population exhibited a more unified sequence for *rhcga*, which suggested that some evolutionary selection pressures had affected this gene. Based on the smaller population (lower nucleotide diversity in ALK population) and adaptation to an extreme alkali environment, we proposed these conservatively nonsynonymous SNPs were the genetic evolution imprints of selective sweep and genetic drift. Because, we detected two convergent evolution amino acid mutation sites in *rhcga* among several alkali environment–adapted Cypriniformes fish, which indicated these mutations may increase the ammonia emission capacity of Rhcga protein. These convergent substitutions at the same loci in different species that have adapted to similar aquatic conditions implied that the more highly conserved Rhcga sequences in alkaline water populations were likely due to strict sweep selection. Converse, the remaining mutations were most likely left over due to random genetic drift. Our results suggested that the most likely pathway for teleost fish in Lake Dali Nur to excrete fatal ammonia is active ion transport, and different species may retain convergent mutations to deal with similar environmental pressures.

In addition to genes mentioned above, we noted that several GO terms are not obviously linked with alkaline tolerance (fig. 2*E*). It is likely that some of these signals are the result of drift or of linkage with genes under selection. For *L. waleckii* in Lake Dali Nur, they must migrate to fresh water river for spawning every year. During the migration, Amur ide face fast and extraordinary environmental changes and stresses, including alkalinity, salinity, temperature, and energy metabolism. Besides, some genes related to sexual hormone release and rhythm regulation may be selected during this process. In our research, we identified several genes related to reproduction and rhythm regulation, such as *gmrhr2* and *mtnr1a* (fig. 2*E* and supplementary table S49, Supplementary Material online). Recently, several studies revealed that some fish species living in extreme environments will invoke some immune-related genes to fight against with extreme environments in the long term (Liang et al. 2015; Tong et al. 2015; Wang et al. 2021). In our research, we also identified many genes were selected in Lake Dali Nur *L. waleckii*, which are related to the immune system (fig. 2*E* and supplementary tables S47 and S48, Supplementary Material online). These genes may enhance the ability of cellular protective response to possible tissue damage caused by extreme alkaline environments.

## Conclusion

The adaptation of *L. waleckii* to an alkaline lake represents the remarkable adaptability of a species to an alkaline environment. We developed a chromosome-level genome of *L. waleckii* inhabiting an extremely alkaline environment, which provided an important genomic resource for the exploitation of alkaline water fishery resources and adaptive evolution research across teleost fish. Based on comparative genomics, several specific characteristics of adaptive changes in *L. waleckii* regarding gene expansion, transposable elements, and selection pressures were detected. Based on the resequencing of 85 *L. waleckii* individuals from divergent populations, genome scans further revealed historical population size fluctuations associated with lacustrine areas and the significant selective sweep regions of Lake Dali Nur *L. waleckii*. These regions harbored a set of candidate genes involved in hypoxia tolerance, ion transport, acid–base regulation, and nitrogen metabolism. In particular, several alkali population–specific amino acid mutations were identified in CA15 gene copies. In addition, two convergent evolution amino acid mutation sites were detected in *rhcga* in several alkali environment–adapted Cypriniformes fish. This study has expanded our understanding of the genetic background of adaptive evolutionary in Cypriniformes fish under extreme alkaline environments and provided a new model example for exploring the convergent evolution mechanism of different species under the same habitat.

## Materials and Methods

### Sample Collection

A healthy female *L. waleckii* was collected from Lake Dali Nur, Inner Mongolia (43°22′43′N, 116°39′24′E) (supplementary fig. S1, Supplementary Material online); fresh muscle was immediately frozen in liquid nitrogen for 20 min and then stored at −80 °C for DNA sequencing. Besides, 25 *L. waleckii* individuals were collected from Lake Dali Nur (DL), Inner Mongolia, 13 individuals were collected from the WS, 7 individuals were collected from the HL, and 12 individuals were collected from the YD. The fins of these individuals were stored at anhydrous ethanol for DNA extraction.

### Genome Sequencing, Assembly, and Annotation

See Supplementary File 1 for the detailed method of genome sequencing, assembly, and annotation.

### Evolutionary and Comparative Genomic Analyses

We used the protein-coding genes of *C. idella*, *A. nigrocauda*, and *L. waleckii* for genomic collinearity analysis by jcvi (v. 1.2.10). Eight species (*A. nigrocauda*, *C. idella*, *L. waleckii*, *Labeo rohita*, *Onychostoma macrolepis*, *Danio rerio*, *T. tibetana*, and *T. dalaica*) were used in the comparison analysis. RepeatModeler was firstly used to detect repeat sequences in these genomes. TEclass (v. 2.1.3) was used to further annotate unclassified repeats. Then, combined with Repbase (http://www.girinst.org/repbase), a species-specific repeat 230 sequence library was constructed. Finally, RepeatMasker (v. 4.1.0) was utilized to search and classify repeats based on this library.

Single-copy genes in *L. waleckii* and nine related species (*T. tibetana*, *T. dalaica*, *D. rerio*, *L. rohita*, *O. macrolepis*, *C. idella*, *Anabarilius grahami*, *A. nigrocauda*, and *Oryzias latipes*) were identified based on gene families constructed from protein sequences of all species employing OrthoFinder (v. 2.5.4) (Emms and Kelly 2019) software. Single-copy orthologous proteins were aligned with MUSCLE (v. 3.8.31). A combined continuous ultralong sequence was constructed from all the translated coding DNA alignments for minimum evolution (ME) phylogenetic tree construction using RAxML. The divergence time was estimated using MCMCTREE (PAML package) (Yang 1997) based on the molecular clock data of the TimeTree database, which the priors set as the *O. latipes* and ancestral of Cypriniformes divided at 230 Ma. The expansion and conversion gene families of *L. waleckii* were identified by CAFÉ (v. 4.2).

To identify the positive selection and rapidly evolution genes in *L. waleckii*, we used BLAST to obtain 10,660 reciprocal best hit (RBH) homologues among *A. nigrocauda*, *C. idella*, *D. rerio*, *L. waleckii*, and *O. macrolepis* (BLAST *E*-value cut-off of 1*e*^−5^). We employed the software PRANK-MSA (v140110) (Loytynoja 2014) with the following parameters, gaprate = 0.025 and gapext = 0.75 to generate coding sequence alignment for each homologous group. To examine the selective constraints on the genes, we estimated the dN/dS ratio (*ω*) using PAML (v4.4b) (Yang 1997). The detailed method for identifying the positive selection and rapidly evolution genes is listed in Supplementary File 1.

### Resequencing and Population Genetic Analysis

The resequencing libraries were constructed by TruePrep DNA Library Prep Kit V2 for Illumina (Vazyme Biotec, Nanjing, China). Whole-genome resequencing was performed using the Illumina Novo Seq 6000 platform. Paired-end reads were aligned to the reference genome using BWA. GATK v4.0.5.2 was employed to genotype all individuals under standard procedures (McKenna et al. 2010). Finally, VCFTOOLS v0.1.06 was used to strictly filter low-quality sites (Danecek et al. 2011).

Based on the SNPs and INDELs, a maximum likelihood tree was constructed by RAxML v8.2.12 (Stamatakis 2014). The PCA and structure analysis were performed using GCTA v1.26.0 and Admixture v1.3.0 with all SNPs (Alexander et al. 2009; Yang et al. 2011). After excluding YD and HL due to their insufficient sample size, the recent demographic history of the DL and WS populations was inferred by the trend in effective population size (Ne) changes using smc++ with default parameters (Patton et al. 2019). Each generation was set to 3 years based on the age at sexual maturity of *L. waleckii*.

### Calculation of the Recombination Rate, *π* Ratio, *F*_st_, and Tajima's *D* and the Identification of Selective Signatures

The recombination rates of ALK and FW were caudated by a R package (FastEPRR) with a sliding window of 20 kb. To investigate the selection signals for adaptability to extreme alkaline environments in the ALK population, we first scanned the genome using *F*_st_ and *π* ratios with a sliding window size of 20 kb and a step size of 10 kb. The average of the *π* ratio values for Dali Nur and the FW river ($\bar{\pi D/\pi F}$) was used to represent the difference in nucleotide diversity. We identified the regions with the 1% highest *F*_st_ values (*F*_st_ > 0.476) or 1% significant differences in nucleotide diversity (|Log2($\bar{\pi D/\pi F}$)| > 1.875). All candidate genes were annotated by blasting candidate regions to the NCBI database. Fine mapping and scanning were performed using *F*_st_, the *π* ratio, and Tajima's *D* with a sliding window size of 10 kb.

### Differential Gene Expression Analysis

See Supplementary File 1 for the detailed method of differential gene expression analysis.

### Gene Family Analysis

All available CA and RH protein genes in *Homo sapiens*, *Mus musculus*, *Gallus gallus*, *Xenopus tropicalis*, *D. rerio*, *G. aculeatus*, *O. niloticus*, *Takifugu rubripes*, *O. latipes*, *A. nigrocauda*, *C. auratus*, *C. idella*, *O. macrolepis*, *T. dalaica*, and *T. tibetana* were downloaded from Ensembl and the public database GenBank. Furthermore, 20 zebrafish genes were used as queries to search against all available genomics resources by TBLASTN and BLASTP, to acquire the candidate genes and proteins. All CA genes and RH genes were aligned using ClustalW with default parameters. We used the maximum likelihood (ML) method to construct the phylogenetic tree. Then, the nomenclature of all these genes was renamed based on their orthologous genes and their phylogenetic position. The conserved motifs of the CA and RH gene family proteins were analyzed using the MEME tool (http://meme-suite.org/). The 3D model of selected genes of *L. waleckii* was predicted by SwissModel.

## Supplementary Material

evad082_Supplementary_Data

## Data Availability

All sequencing data involved in the article have been deposited at the National Genomics Data Center under Bioproject PRJCA009202 and National Center for Biotechnology Information under Bioproject PRJNA298374. The genome of *L. waleckii* and annotation file can be downloaded on the figshare under https://doi.org/10.6084/m9.figshare.20103746.

## Code Availability

All code availability has been listed in the Supplementary File 1.
