# Supplementary material for: The Adaptive Evolution of Leuciscus waleckii in Lake Dali Nur and Convergent Evolution of Cypriniformes Fishes Inhabiting Extremely Alkaline Environments
Source: Genome Biol Evol. 2023 May 17;15(5):evad082. doi: 10.1093/gbe/evad082 (PMC10210623; doi:10.1093/gbe/evad082)
Supplement: evad082_Supplementary_Data [file evad082_supplementary_data.zip › Supplementary File1.docx]

**The chromosome-level genome of *Leuciscus waleckii* provide insight into the adaptive and convergent evolution under the extreme alkaline environment in Lake Dali Nur**

Zhixiong Zhou^1,2,3^, Junyi Yang^1,2,3^, Hongzao Lv^1,2,3^, Tao Zhou^1,2,3^, Ji Zhao^1,2,3^, Huaqiang Bai^1,2,3^, Fei Pu^1,2,3^, Peng Xu^1.2.3^

1 State Key Laboratory of Marine Environmental Science, College of Ocean and Earth Sciences, Xiamen University, Xiamen, 361102, China

2 Fujian Key Laboratory of Genetics and Breeding of Marine Organisms, College of Ocean and Earth Sciences, Xiamen University, Xiamen 361102, China

3 Laboratory for Marine Biology and Biotechnology, Pilot National Laboratory for Marine Science and Technology, Qingdao, 266071, China

Corresponding author: Peng Xu, E-mail: [xupeng77@xmu.edu.cn](mailto:xupeng77@xmu.edu.cn)

**SUPPLEMENTARY INFORMATION**

**Table of Contents**

[Methods 6](#_Toc129976153)

[Code Availability 9](#_Toc129976154)

[Genome assembly and annotation 12](#_Toc129976155)

[Supplementary Figure 13](#_Toc129976156)

[Fig S1. Photo of *L. waleckii* 13](#_Toc129976157)

[Fig S2. Genome size estimation. 14](#_Toc129976158)

[Fig S3. The comparison of gene structure elements between Amur Ide and related species. 15](#_Toc129976159)

[Fig S4. Gene structure and function annotation. 16](#_Toc129976160)

[Fig S5. The Hi-C heatmap of Amur Ide represented the assembly of the chromosome. 17](#_Toc129976161)

[Fig S6. The genome landscape of Amur Ide and the genomic collinearity between Amur Ide and Zebrafish. 18](#_Toc129976162)

[Fig S7. Genomic collinearity of L. waleckii and its related species 19](#_Toc129976163)

[Fig S8. The divergence distribution of TEs in the Amur Ide and related species. 20](#_Toc129976164)

[Fig S9. The divergence distribution of LTR and LINEs in the Amur Ide and related species. 21](#_Toc129976165)

[Fig S10. The bubble diagram of GO enrichment of expansion gene families in Amur Ide. 22](#_Toc129976166)

[Fig S11. The bubble diagram of GO enrichment of contraction gene families in Amur Ide. 23](#_Toc129976167)

[Fig S12. The volcanic map and the heatmap of differential expression analysis in the gill, liver and kidney of Amur Ide. 24](#_Toc129976168)

[Fig S13. The population genetic of the *L. waleckii* populations. (A) Maximum likelihood tree of the relationships between the 85 *L. waleckii* samples based on SNPs. Individuals from different populations are represented by different colours. (B) 3D plot visualizing the principal component analysis (PCA). 25](#_Toc129976169)

[Fig S14*.* (A) Distribution of monsoon in Northeast Asia and the location of Lake Dali Nur; (B) The schematic diagram of the drainage area of the ancient Paleolake and current Lake Dali Nur 26](#_Toc129976170)

[Fig S15. The enrichment of candidate selected genes. 27](#_Toc129976171)

[Fig S16. The venn diagram of number of genes among DEGs and candidate selected genes. 28](#_Toc129976172)

[Fig S17. The phylogenetic tree of CA genes between Amur Ide and releted species. 29](#_Toc129976173)

[Fig S18. The motif of 19 CA genes in Amur Ide. 30](#_Toc129976174)

[Fig S20. The phylogenetic tree of RH glycoproteins between Amur Ide and releted species. 32](#_Toc129976175)

[Fig S21. The motif of 7 RH genes in Amur Ide. 33](#_Toc129976176)

[Fig S22. The 3D structure of RHCG-a of ALK and FW *L. waleckii* population. 34](#_Toc129976177)

[Supplementary Table 35](#_Toc129976178)

[Table S2. The statistics of 17 K-mer analysis of *L. waleckii*. 36](#_Toc129976179)

[Table S3. The statistics of PacBio Sequencing. 37](#_Toc129976180)

[Table S4. The statistics of assembly of Amur Ide genome. 38](#_Toc129976181)

[Table S5. The statistics of Repeat elements annotation of Amur Ide genome. 39](#_Toc129976182)

[Table S6. Detailed classification of repeat sequences in Amur Ide genome. 40](#_Toc129976183)

[Table S7. The statistics of gene structure annotation of Amur Ide genome. 40](#_Toc129976184)

[Table S8. The statistics of gene function annotation of Amur Ide genome. 41](#_Toc129976185)

[Table S9. The statistics of chromosome assembly of Amur Ide. 42](#_Toc129976186)

[Table S10. The BUSCO survey of Amur Ide. 43](#_Toc129976187)

[Table S11. The mapping statistics of Illumina sequencing reads to reference Amur Ide genome. 44](#_Toc129976188)

[Table S12. The statistics of SNPs in the genome sequencing individual. 45](#_Toc129976189)

[Table S13. The statistics of orthologues gene families between Amur Ide and related species. 46](#_Toc129976190)

[Table S14. The statistics of detailed classification of transposable element sequences between Amur Ide and related species*.* 46](#_Toc129976191)

[Table S15. The statistics of expansion and contraction gene families between *L. waleckii* and related species. 47](#_Toc129976192)

[Table S16. The GO enrichment of expansion gene families in Amur Ide*.* 48](#_Toc129976193)

[Table S17. The GO enrichment of contraction gene families in Amur Ide. 48](#_Toc129976194)

[Table S18. The gene numbers of GGT gene family between Amur Ide and related species. 48](#_Toc129976195)

[Table S19. The rapid evolution genes and function annotation in Amur Ide. 49](#_Toc129976196)

[Table S20. The GO enrichment of rapid evolution genes in Amur Ide. 49](#_Toc129976197)

[Table S21. The positively selected genes and function annotation in Amur Ide. 49](#_Toc129976198)

[Table S22. The GO enrichment of positively selected genes in Amur Ide. 49](#_Toc129976199)

[Table S23. The statistics of ReviGO analysis of rapid evolution genes in Amur Ide. 49](#_Toc129976200)

[Table S24. The statistics of ReviGO analysis of positively selected genes in Amur Ide. 49](#_Toc129976201)

[Table S25. The statistics of RNA sequencing between ALK and FW Amur Ide population. 50](#_Toc129976202)

[Table S26. The statistics of gene expression in gill between ALK and FW Amur Ide population. 51](#_Toc129976203)

[Table S27. The up-regulated gene list of ALK Amur Ide population in gill. 51](#_Toc129976204)

[Table S28. The down-regulated gene list of ALK Amur Ide population in gill. 51](#_Toc129976205)

[Table S29. The statistics of gene expression in kidneys between ALK and FW Amur Ide population. 51](#_Toc129976206)

[Table S30. The up-regulated gene list of ALK Amur Ide population in the kidney. 51](#_Toc129976207)

[Table S31. The down-regulated gene list of ALK Amur Ide population in the kidney. 51](#_Toc129976208)

[Table S32. The statistics of gene expression in liver between ALK and FW Amur Ide population. 51](#_Toc129976209)

[Table S33. The up-regulated gene list of ALK Amur Ide population in the liver. 51](#_Toc129976210)

[Table S34. The down-regulated gene list of ALK Amur Ide population in the liver. 51](#_Toc129976211)

[Table S35. The GO enrichment of up-regulated gene of ALK Amur Ide population in gill. 51](#_Toc129976212)

[Table S36. The GO enrichment of down-regulated gene of ALK Amur Ide population in gill. 51](#_Toc129976213)

[Table S37. The GO enrichment of up-regulated gene of ALK Amur Ide population in kidney. 52](#_Toc129976214)

[Table S38. The GO enrichment of down-regulated gene of ALK Amur Ide population in kidney. 52](#_Toc129976215)

[Table S39. The GO enrichment of up-regulated gene of ALK Amur Ide population in liver. 52](#_Toc129976216)

[Table S40. The GO enrichment of down-regulated gene of ALK Amur Ide population in liver. 52](#_Toc129976217)

[Table S41. The rapid evolution genes with differential expression in ALK population. 52](#_Toc129976218)

[Table S42. The positively selected genes with differential expression in ALK population. 52](#_Toc129976219)

[Table S43. The statistics of Genome Resequencing between ALK and FW Amur Ide population. 53](#_Toc129976220)

[Table S44. The statistics of SNPs between 4 Amur Ide population. 53](#_Toc129976221)

[Table S45. The statistics of Fst and π ratio scanning in 25 chromosomes between ALK and FW Amur Ide population. 54](#_Toc129976222)

[Table S46. The candidate selected regions that were identified by Fst in ALK Amur Ide population. 55](#_Toc129976223)

[Table S47. The candidate genes that were identified by Fst in ALK Amur Ide population. 55](#_Toc129976224)

[Table S48. The candidate selected regions that identified by π ratio in ALK Amur Ide population. 55](#_Toc129976225)

[Table S49. The candidate genes that were identified by π ratio in ALK Amur Ide population. 55](#_Toc129976226)

[Table S50. The GO enrichment of candidate selected genes in ALK Amur Ide population. 55](#_Toc129976227)

[Table S51. The KEGG enrichment of candidate selected genes in ALK Amur Ide population. 55](#_Toc129976228)

[Table S52. The copy numbers of CA genes between Amur Ide and related species. 56](#_Toc129976229)

[Table S53. The accession number of CA genes in Cyprinidae fish. 56](#_Toc129976230)

[Table S54. The statistics of 19 CA genes in ALK Amur Ide population. 56](#_Toc129976231)

[Table S55. The statistics of highly differentiated sites in CA genes between ALK and FW Amur Ide population. 56](#_Toc129976232)

[Table S56. The accession number of RH genes in Cyprinidae fish. 56](#_Toc129976233)

[Table S57. The statistics of 7 RH genes in ALK Amur Ide population. 56](#_Toc129976234)

[Table S58. The statistics of highly differentiated sites in *rhcga* between ALK and FW Amur Ide population. 56](#_Toc129976235)

# Methods

**Genome sequencing**

The high-molecular-weight (HMW) genomic DNA (gDNA) for PacBio and Hi-C was extracted by AMPure XP beads (Beckman Coulter, High Wycombe, UK) under previous method ^1^. Meanwhile, normal-molecular-weight (NMW) gDNA for Illumina was extracted using the PureLink Genomic DNA Mini Kit (hermo Fisher Scientific, Shanghai, China). Nucleic acid concentrations were quantified by the Qubit fluorometer 4.0 (Thermo Fisher Scientific, Waltham, MA), and integrity was checked by Agilent2100 Bioanalyzer (Agilent Technologies, Palo Alto, Calif). According to Illumina standard operating procedures, a shotgun library with 350 bp insert size was constructed. Illumina raw reads were generated from Hiseq-X Ten platform, after which uncertain bases (represented by “N”) and low-quality reads (Q < 5) were trimmed by SolexaQA++ ^2^. Finally, all clean reads were retained for the evaluation of genome size and polishing of preliminary contigs. The SMRTbell large fragment sequencing library was constructed according to standard procedures provided by Pacific Biosciences and sequenced on PacBio Sequel Ⅱ platform. *Mbol* restriction enzyme was used to digest the HMW gDNA after fixing conformation of HMW gDNA by formaldehyde, followed by repair the 5′ overhangs with biotinylated residues. The isolated DNA was reverse-crosslinked, purified and filtered for biotin-containing fragments after the ligation of blunt-end in situ. Thereafter, the DNA was sheared into fragments by ultrasonic, the sheared DNA ends was subsequently repaired by T4 DNA polymerase, T4 polynucleotide kinase and Klenow DNA polymerase. Then, a dATP was attached to the 3' ends of the end-repaired DNA and 300-500 bp fragments was retrieved by Caliper LabChip Xte (PerkinElmer, USA). The DNA concentration was determined by Qubit and the Illumina Paired End adapters was ligated to the DNA by T4 DNA Ligase. Thereafter, 12 cycles PCR reactions was conducted and products were purified by AMpureXP beads. Finally, sequencing of Hi-C library was performed on Illumina Hiseq X-Ten platform and yielded a total of 102.93 Gb pair-end raw reads and 100.36 Gb was retained after quality control (Table S1).

**Genome assembly and annotation**

Reads from the three types of libraries were used in different assembly stages separately. Illumina sequencing data were used for genome survey, PacBio sequencing data were served in contig assembly, and Hi-C reads were used in chromosome-level scaffolding. In genome survey, the 17 mers frequency of 73 Gb Illumina clean data was counted with 1 bp sliding window by Jellyfish (Table S1 and Fig S2)^3^. Finally, the proportion of heterozygosity in *L. waleckii* genome was evaluated as 0.56%, and the genome size was estimated as 1125.03 Mb, with a repeat content of 57.61% (Table S2). A total of 62 Gb raw reads were generated by PacBio with a mean insert size of 35,943, resulting in ~54.87X coverage of the k-mer estimated genome size of *L.waleckii* (Table S3). Long reads generated from the PacBio SEQUEL platform were subsequently processed by a self-correction of errors using Canu^4^. Based on Overlap-Layout-Consensus algorithm, we detected overlaps from input reads and assembled the final String Graph by wtdbg2 ^5^. Subsequently we used FALCON-unzip pipeline to generate phased contig sequences for further calling highly accurate consensus sequences using variantCaller in GenomicConsensus package, which was employed arrow algorithm, and polishing the contigs using Illumina reads by pilon^6^.

For chromosome-level scaffolding, we first filtered Hi-C reads with the same protocol as Illumina reads. Subsequently, we mapped the Hi-C clean reads to the *de novo* assembled contigs by BWA^7^ with default parameter. We removed the reads that unmapped within 500bp of a restriction enzyme site. Using 3D-DNA, we assembled the chromosome-level scaffolding based on the signal of genomic proximity in Hi-C data sets. In this stage, all parameters were default.

Repetitive sequences of the *L. waleckii* genome were annotated using both homology-based search and de novo methods. Combined with Repbase (v. 20,181,026; http://www.girinst.org/repbase), a repeat sequence library was constructed. RepeatMasker (v. 4.1.0) were utilized to search and classify repeats based on this library. TEclass (v. 2.1.3)^8^ was used to further annotate unclassified repeats. The built-in script buildSummary.pl from RepeatMasker (v. 4.1.0) was used to summarize Transposable Elements (TEs) annotation results. Then two scripts, calcDivergenceFromalign.pl and createRepeatLandscape.pl, were used to calculate the Kimura divergence value and draw repeated landscapes. The nucleotide distances between all copies of each TE measured using the Kimura two-parameter method were compared to estimate insertion age ^9^. Tandem Repeats Finder (v. 4. 09)^10^ was used to identify tandem repeats. All repetitive regions except tandem repeats were soft-masked for protein-coding gene annotation. For gene structure prediction, we used both homology-based and de novo strategies to predict genes in the *L. waleckii* genome. For homology-based prediction, we mapped the protein sequences of *Cyprinus carpio^11^*, *Danio rerio*^12^, *Ctenopharyngodon idellus*^13^, *Sinocyclocheilus grahami*^14^ and *L. waleckii*^15^ onto the generated assembly using BLAT^16^ (version 35) with an e-value ≤ 1e-5. Then, we used GeneWise^17^ (version 2.2.0) to align the homologous in the *L. waleckii* genome against the other five teleosts for gene structure prediction. In the de novo approach, we used several software packages, including Augustus (version 2.5.5)^18^, GlimmerHMM (version 3.0.1)^19^, SNAP (version 1.0)^20^, Geneid (version 1.4.4)^21^ and GenScan (version 1.0)^22^. In addition, we also used RNA-seq data to predict the structure of transcribed genes using TopHat (version 1.2)^23^ and Cufflinks (version 2.2.1)^24^. Using EvidenceModeler (version 1.1.0) ^25^, we combined the setof predicted genes generated from the three approaches into a non-redundant gene set and then used PASA (version 2.0.2)^26^ to annotate the gene structures. For gene function annotation, we used BLASTP to align the candidate sequences to the NCBI and Swissport protein databases with E values < 1 × 10−5. Then, we performed the functional classification of GO categories with the InterProScan program (version 5.26)^27^ and used KEGG Automatic Annotation Server (KAAS)^28^ to conduct the KEGG pathway annotation analysis. The programs tRNAScan-SE (v. 1.3.1) and RNAmmer (v. 1.2) were used to predict tRNA and rRNA, respectively. The other ncRNAs were identified by searching against the Rfam database (<http://eggnogdb.embl.de/>).

Assembly completeness and accuracy were evaluated by multiple methods. First, the Illumina short reads were remapped to the genome using BWA (v. 0.7.17). Then, we used the Benchmarking Universal Single Copy Orthologues (BUSCO) to test the integrality of the final assembly and the lineage dataset was actinopterygii_odb10.

**Identification of positive selection genes and rapidly evolution genes**

We used three hypotheses: 1) H0, all branches have the same ω; 2) H1, the branch leading to sunfish has a different ω, whereas the other branches have the same ω; and 3) H2, all branches have independent ω. We used likelihood values and degrees of freedom of the three hypotheses to perform a likelihood-ratio test (LRT). We selected genes whose likelihood values of H1 were significantly larger (adjusted LRT p value of < 0.05) than those of H0, and genes whose likelihood values of H2 are not significantly larger than those of H1. Finally, we identified 369 fast-evolving genes with significant false discovery rate (FDR)-corrected p values (<0.05) in *L. waleckii.*

In addition, we also ran branch-site models (model = 2; NSsite = 2) to detect the genes with positively selected sites in *L. waleckii*. For the null hypothesis, we set ‘fix_omega = 1; omega = 1’, whereas for the alternative hypothesis, we set ‘fix_omega = 0; omega = 1.5’ with the tree ‘((*A. nigrocauda*, (*C. idella*, *L. waleckii #1*)), *O. macrolepis*, *D. rerio*)’. Using an FDR-corrected LRT p value (adjusted LRT p value) cut-off of 0.05, we identified 131 positively selected genes in *L. waleckii*.

**Differential gene expression analysis**

For RNA sequencing, three tissues (gill, kidney and liver) were dissected and collected from three Lake Dali Nur *L. waleckii* individuals and three Wusuli River *L. waleckii* individuals, which are biological replicates. Total RNA was extracted from the 3 tissues using TRIzol (Invitrogen, Carlsbad, CA, USA). The high-quality RNA samples were sequenced on Illumina HiSeq 2000 platforms according to the manufacturer’s instructions. After sequencing, the adaptor sequences and low-quality reads (quality score ≤ 20) were eliminated to obtain high-quality clean reads using SolexaQA; these reads were then aligned to a reference genome of *L. waleckii* using HISAT2^29^. Stringtie^30^ was used to assemble genes and identify differentially expressed genes (DEGs). We measured the gene expression level based on the fragments per kilobase of exon model per million mapped reads (FPKM). Genes with expression differences fulfilling statistical significance criteria (q-value < P value, P value < 0.05, |log2 (fold change)| > 2) were regarded as differentially expressed genes. To understand the functions of these DEGs, Gene Ontology (GO) functional enrichment and Kyoto Encyclopedia of Genes and Genomes (KEGG) pathway analyses were carried out using OmicShare tools (www.omicshare.com/tools). The threshold for significant enrichment of gene sets was set as p < 0.05.

# Code Availability

The versions, settings and parameters of the software used in this work are as follows:

Genome assembly:

1) Wtdbg2: version 2.5; parameters: -g=1125m, other parameters were set as default; 2) Canu+-: version: 2.1.0; parameters: genomeSize=1125m, minReadLength=2000, minOverlapLength=500, corOutCoverage=120, corMinCoverage=2; 3) pilon: version:1.22; all parameters were set as default; 4) 3D-DNA: parameters: all parameters were set as default.

Genome annotation:

1) RepeatMasker: version: open-4.0.7; parameters: -a -nolow -no_is -norna -parallel 1; 2) RepeatModeler: version: open-1.0.10; parameters:-database genome -engine ncbi -pa 15; 3) LTR_FINDER: version:1.05; parameters: -C -w 2; 4) RepeatScout: version: 1.0.5; parameters: all parameters were set as default; 5) TEclass: version: 2.1.3; parameters: all parameters were set as default; 6) TRF: matching weight = 2, mismatching penalty = 7, INDEL penalty = 7, match probability = 80, INDEL probability = 10, minimum alignment score to report = 50, maximum period size to report = 2000, -d –h; 7) Augustus: version:3.1.2; parameters: --extrinsicCfgFile --uniqueGeneId=true --noInFrameStop=true --gff3=on --genemodel=complete --strand=both; 8) GlimmerHMM: version:3.0.3; parameters: -f –g; 9) Genscan: -cds; 10) Geneid: version: 1.2; parameters: -P -v -G -p geneid; 11) Genewise: version: 2.4.0; parameters: -trev -genesf -gff –sum; 12) BLAST: version 2.7.1; parameters: -p tblastn -e 1e-05 -F T -m 8 -d; 13) EVidenceModeler: version: 1.1.1; parameters: G genome.fa -g denovo.gff3 –w weight_file -e transcript.gff3 -p protein.gff3 --min_intron_length 20 14) PASA: version: 2.3.3; parameters: all parameters were set as default; 15) tRNAScan-SE: version 1.3.1; parameters: all parameters were set as default;. 16) RNAmmer: version 1.2; parameters: all parameters were set as default.

Evolutionary and comparative genomic analyses:

1)Blastp: parameters: -e 1e-7 -outfmt 6; 2)Orthofinder: parameters: all parameters were set as default; 3) MUSCLE: version 3.8.31; parameters: all parameters were set as default; 4) Gblocks: version: 0.91b; parameters: all parameters were set as default; 5) RAxML: version: 8.2.12; parameters: -n sp -m PROTGAMMAAUTO -T 20 -f a 6) MCMCTREE: parameters: all parameters were set as default 7) CAFÉ: version 4.2; parameters: all parameters were set as default; 8) PRANK-MSA: version 140110; gaprate = 0.025 and gapext = 0.75; 9) PAML: version 4.4b; parameters: For REGs identification: mode = 2; NSsite = 0 Codonfreq = 2; kappa = 2.5; initial omega = 0.2; (1) H0, all branches have the same ω; (2) H1, the branch leading to sunfish has a different ω, whereas the other branches have the same ω; and (3) H2, all branches have independent ω; For PEGs identification: model = 2; NSsite = 2; (1) null hypothesis: fix_omega = 1; omega = 1; (2) alternative hypothesis: fix_omega = 0; omega = 1.5.

Differential gene expression analysis

1) SolexaQA: version 3.1.3; parameters: Q = 20; 2) HISAT2: version 2.2.2; parameters: all parameters were set as default; 3) Stringtie: version 1.3.6; parameters: all parameters were set as default;

Resequencing and population genetic analysis

1) BWA: version 0.7.17; parameters: all parameters were set as default; 2) GATK: version 4.0.5.2; parameters: QD < 5.0 || FS > 35.0 || MQ < 55.0 || SOR > 3.0 || MQRankSum < -12.5 || ReadPosRankSum < -8.0>; 3) VCFTOOLS; version 0.1.06; parameters: -mac 2 -min-alleles 2 -max-missing-count 2; 4) RAxML version 8.2.12; parameters: -n sp -m -m GTRGAMMA -T 20 -# 1000; 5) GCTA; version 1.26.0 parameters: all parameters were set as default; 6) Admixture: version 1.3.0; parameters: all parameters were set as default; 7) smc++: version 1.15.4; parameters: mutation rate and recombination of 2e-9, --knots 150, --em-iteration 500, -g 3.

Calculation of the recombination rate, π ratio, Fst, and Tajima’s D, and the identification of selective signatures

1)VCFTOOLS; version 0.1.06; For Fst: parameters: -fst-window-size 20000 –fst-window-step 10000; For π: parameters: –window-pi 20000 –window-pi-step 10000； For Tajima‘S D: --TajimaD 10000.

Gene family analysis

1) TBLASTN: parameters: -e 1e-7 -outfmt 6; 2) Blastp: parameters: -e 1e-7 -outfmt 6; 3) ClustalW: version 2.1; parameters: all parameters were set as default; 4) RAxML version 8.2.12; parameters: -n sp -m -m GTRGAMMA -T 20 -# 1000; 5) MEME tool: <http://meme-suite.org/>; parameters: all parameters were set as default; 6) SwissModel: <https://swissmodel.expasy.org/>; parameters: all parameters were set as default.

# Genome assembly and annotation

A high-quality chromosome-level genome is needed for the downstream analysis of adaptive microevolution^31^. Using the established method, the genome size was evaluated to be approximately 1,125.03 Mb, the heterozygous rate and repeat rate were evaluated as 0.56% and 57.61% respectively by 17-mer analysis (Fig S2 and Table S3) ^32^. Using the PacBio platform, we sequenced the genomes of Amur Ide in Lake Dali Nur. The assembled genome spanned 1,103 Mb, with a contig N50 length of 1.52 Mb (Tables S4). Genome annotation showed that the Amur Ide genome comprises approximately 49.92% repetitive sequences (Tables S5 and Table S6), which was comparable to the repeat content of other Cypriniform species genomes^11,13,33^. In the Amur Ide assembly, we predicted 27,633 protein-coding genes, of which 96.3% of the protein sequences showed similarity to protein sequences in public databases (Table S7, Table S8, Fig S3 and Fig S4). The contigs were then anchored and oriented into a chromosomal-scale assembly using the Hi-C scaffolding approach. Ultimately, we obtained a draft genome assembly of 1,105 Mb in length, with a scaffold N50 value of 39.64 Mb (Table S9). The genome assembly contained 25 chromosomes, with chromosome lengths ranging from 28.42 to 71.37 Mb, and covered 1,020 Mb (92.32%) of the *L.waleckii* assembly (Table S9, Fig S5 and Fig S6A). BUSCO analysis showed that the assembly retrieved 96.4% of the conserved single-copy orthologous genes (Table S10). In addition, we mapped Illumina short reads to the L. waleckii reference genome with a mapping ratio of 99.05% and generated 2,707,134 SNPs (Table S11, Table S12). This evidence supported the high-quality assembly of the *L. waleckii* genome.

# Supplementary Figure

## Fig S1. Photo ­­­­of *L. waleckii*

**
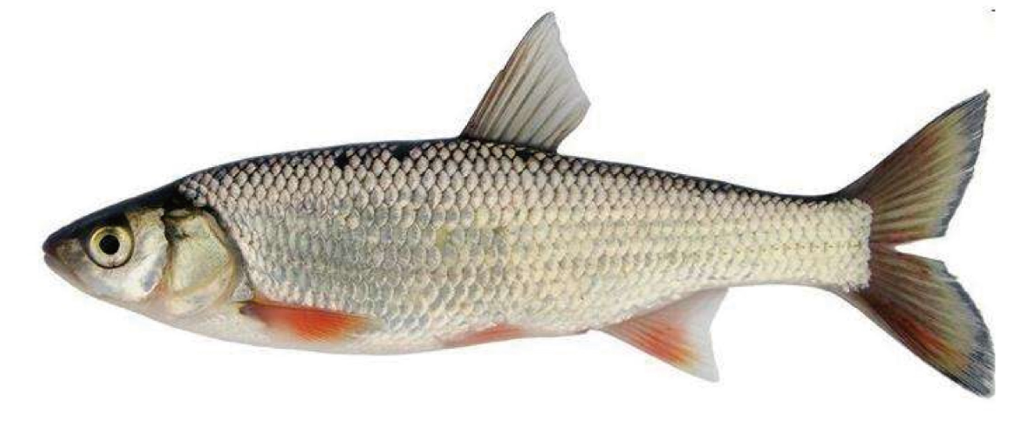

­­­**

## Fig S2. Genome size estimation.


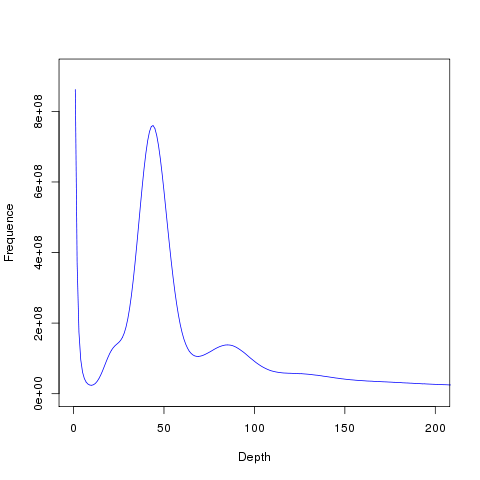


We used 3.7 ×10^9^ sequence reads and obtained 49 × 10^9^ 17-mers. The peak depth is 43. The genome size (G) is correlated with the 17-mer number (N) and the peak of 17-mer frequency (D). Their relationship can be expressed in an empiric formula: G = N / D. The estimated genome size is 1125.03 Mb.

##
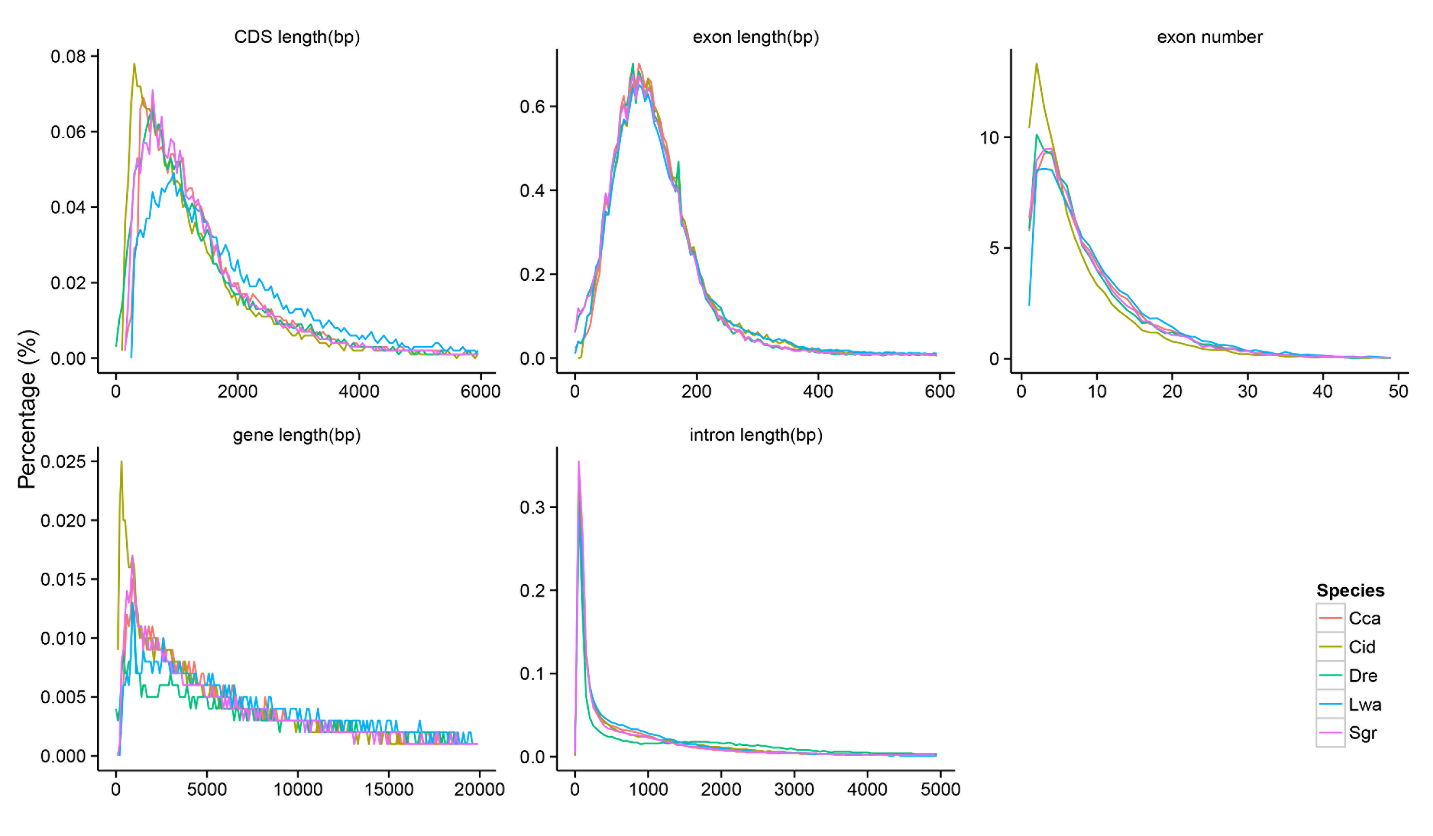
Fig S3. The comparison of gene structure elements between Amur Ide and related species.

##
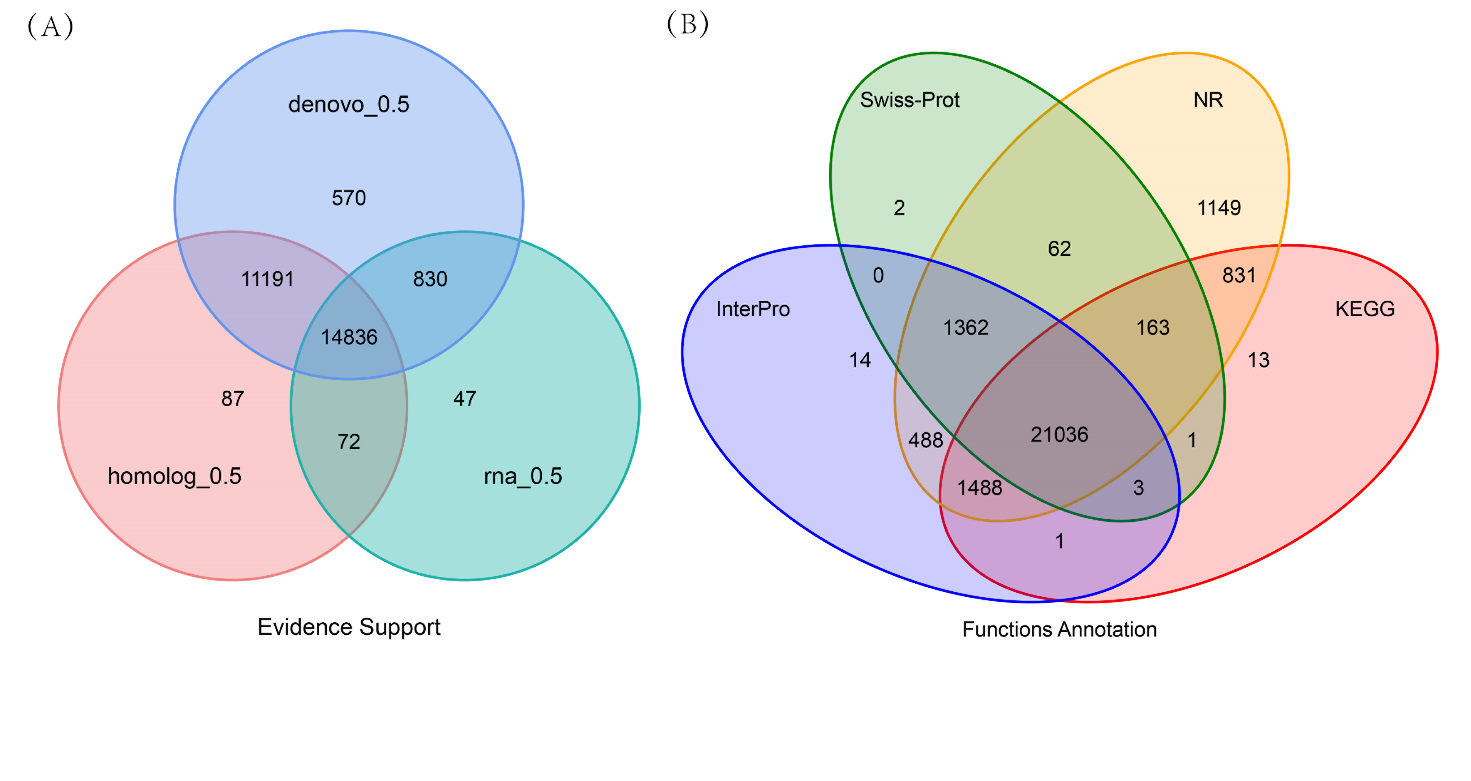
Fig S4. Gene structure and function annotation.

(A) Venn diagram of the number of genes with structure prediction based on different strategies. (B) Venn diagram of the number of functionally annotated genes based on different public databases.

## Fig S5. The Hi-C heatmap of Amur Ide represented the assembly of the chromosome.

**
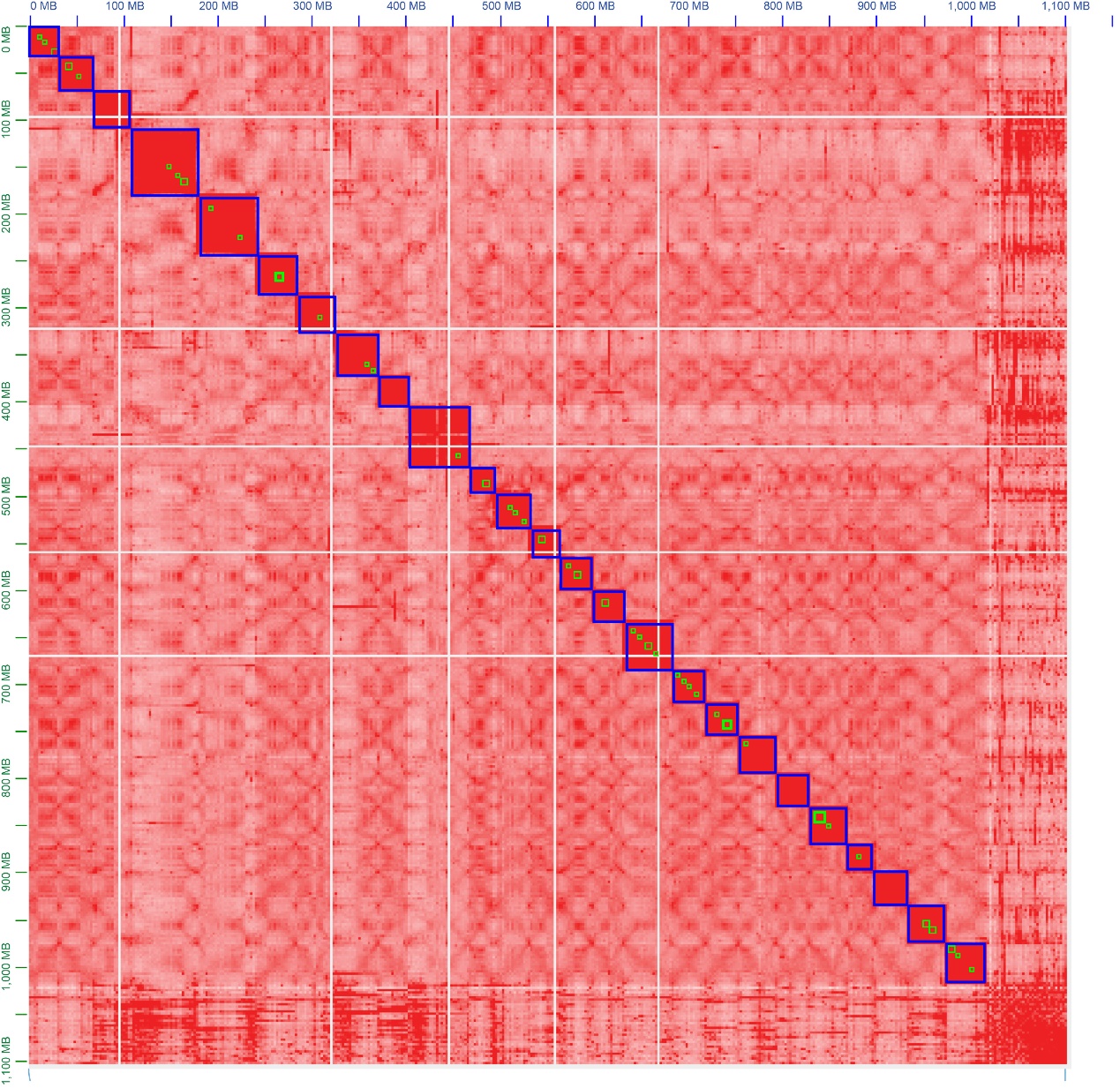
**

Each chromosome is highlight with blue square. Each contig fragments that anchored to the chromosome is represented by green square.

##
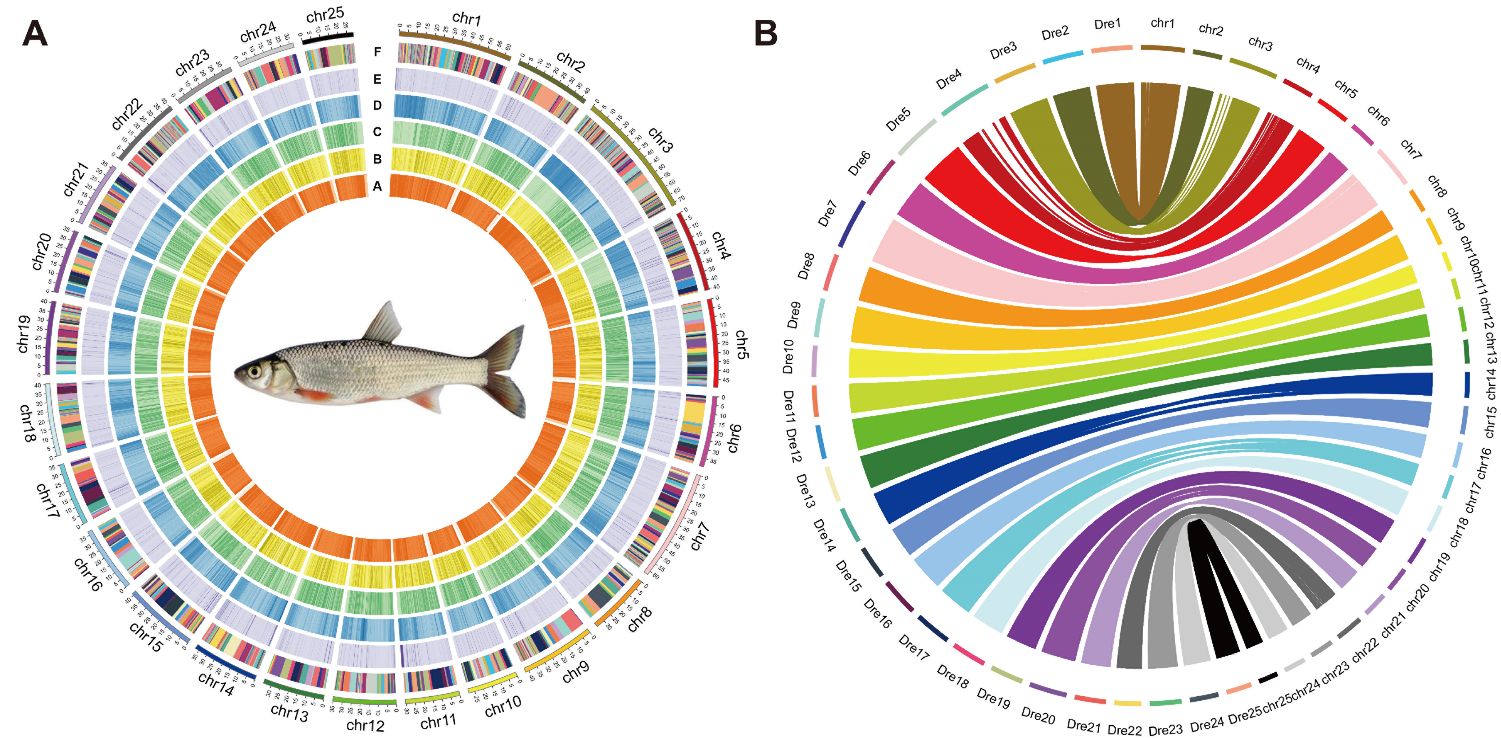
Fig S6. The genome landscape of Amur Ide and the genomic collinearity between Amur Ide and Zebrafish.

**(A) Circos plot of 25 chromosome-level scaffolds, representing annotation results of genes, ncRNAs and transposable elements on these scaffolds.** The tracks from inside to outside are gene abundance of the positive strand (A), gene abundance of the negative strand (B), TE abundance of positive-strand (C), TE abundance of the negative strand (D), the ncRNA abundance of both strands (E), and contigs which comprised the scaffolds (F). **(B)** **Circos diagram between *L. waleckii* and *D. rerio*.** Each colored arc represents a 1 Kb fragment match between two species. We messed up the order of the Amur Ide chromosomes on the image for better illustrate our results.

## Fig S7. Genomic collinearity of L. waleckii and its related species


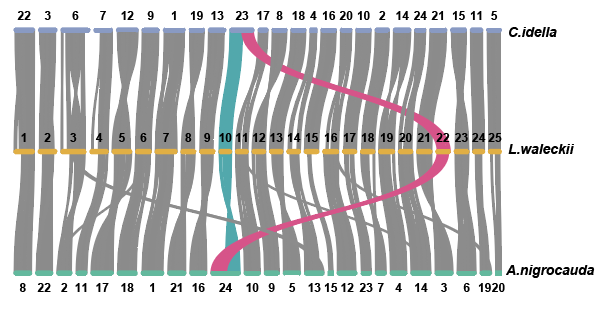


10 and 22 chromosomes are highlighted by different colours.

##
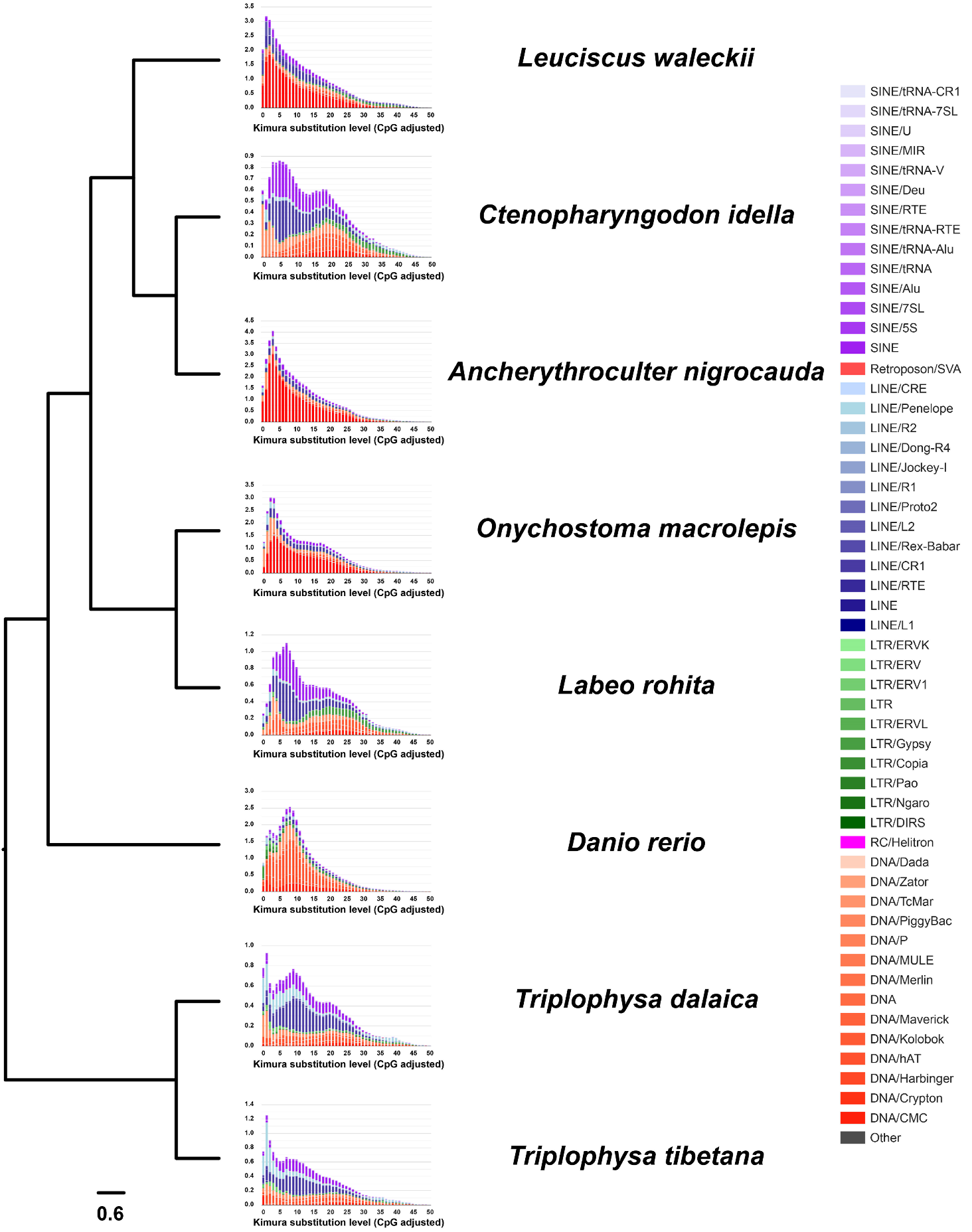
Fig S8. The divergence distribution of TEs in the Amur Ide and related species.

Different types of TEs are represented by different colors, which is listed in color code. The y-axis represents the percentage of genome size. The x-axis represents the kimura substitutions level (CpG adjusted).

## Fig S9. The divergence distribution of LTR and LINEs in the Amur Ide and related species.


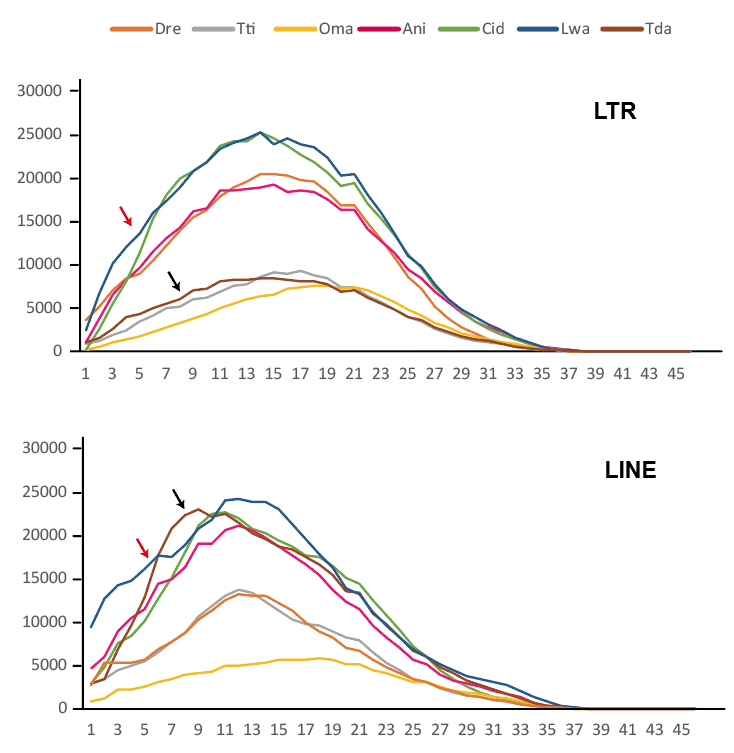


Different species are represented by different colors, which is listed in color code. The y-axis represents the TE copies number. The x-axis represents the kimura substitutions level (CpG adjusted). The red arrow marked the burst of LTR and LINE of *L. waleckii* at K<10, compared to *A. nigrocauda* and *C. Idella*. The black arrow marked the burst of LTR and LINE of *T. dalaica* at K < 14, compare to *T. tibetana*. Dre: *D. rerio*, Tti: *T. tibetana*, Oma: *O. macrolepis*, Ani: *A. nigrocauda*, Cid: *C. idella*, Lwa: *L. waleckii*, Tda: *T.* *dalaica*).

##
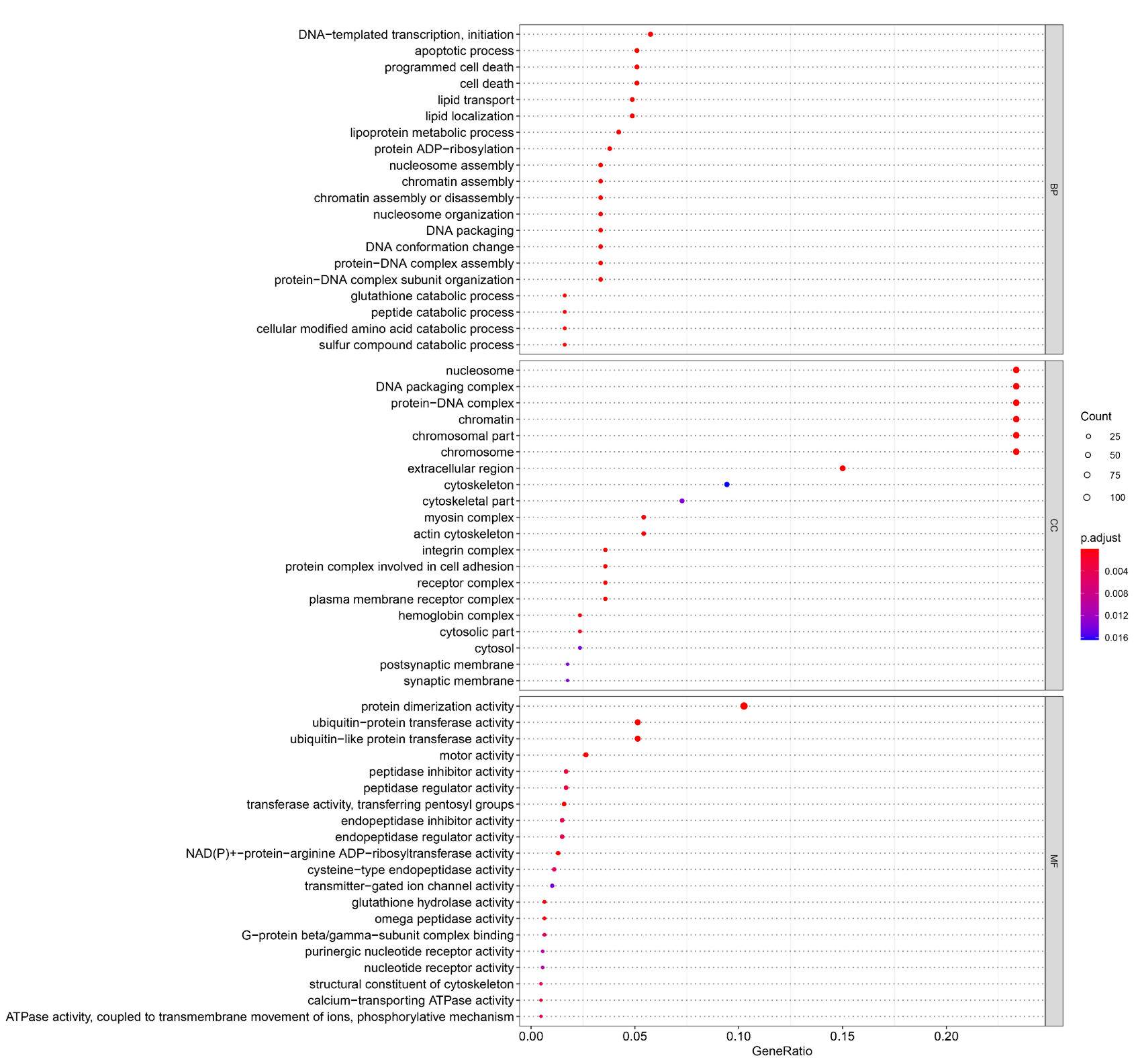
Fig S10. The bubble diagram of GO enrichment of expansion gene families in Amur Ide.

##
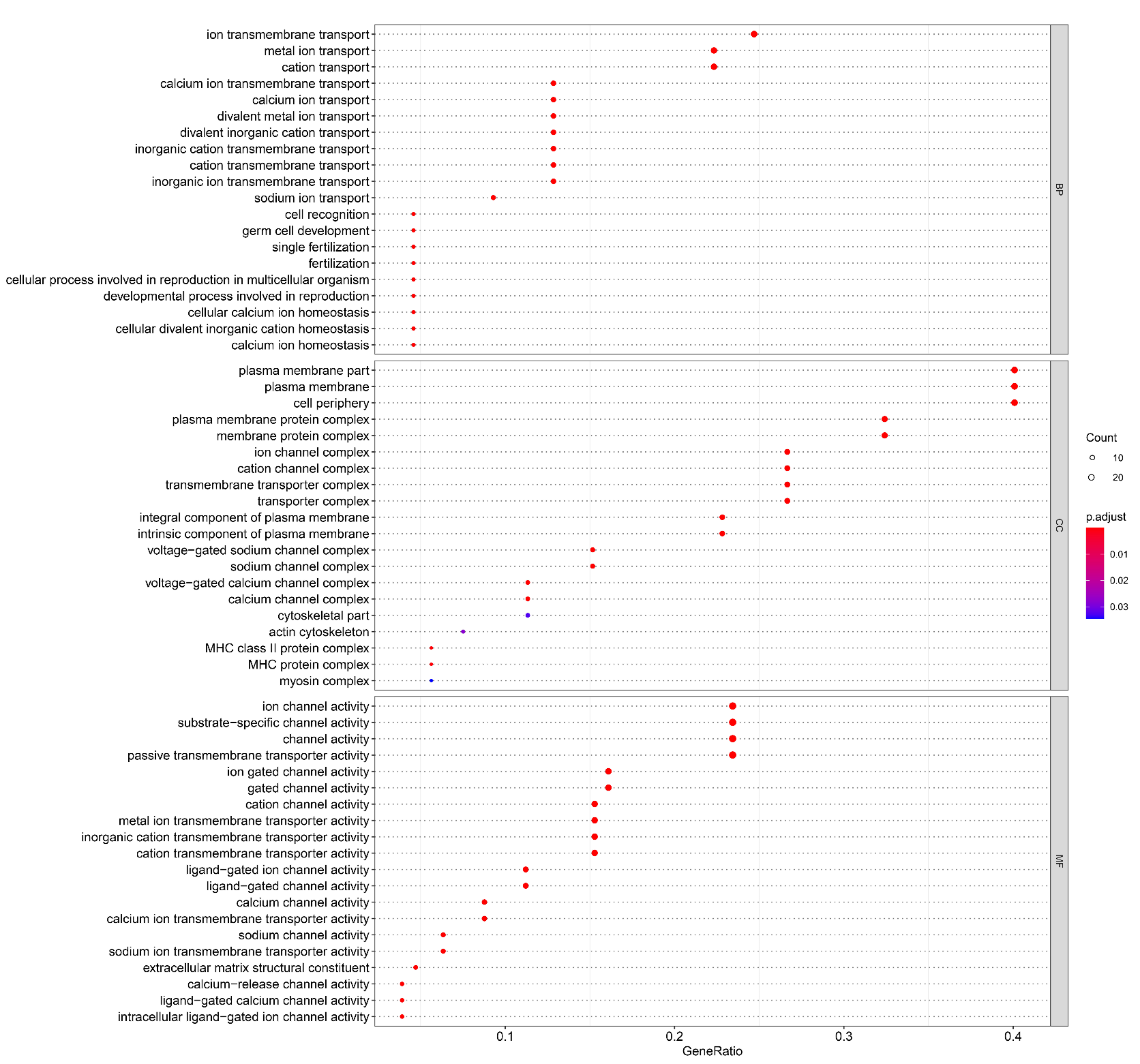
Fig S11. The bubble diagram of GO enrichment of contraction gene families in Amur Ide.

##
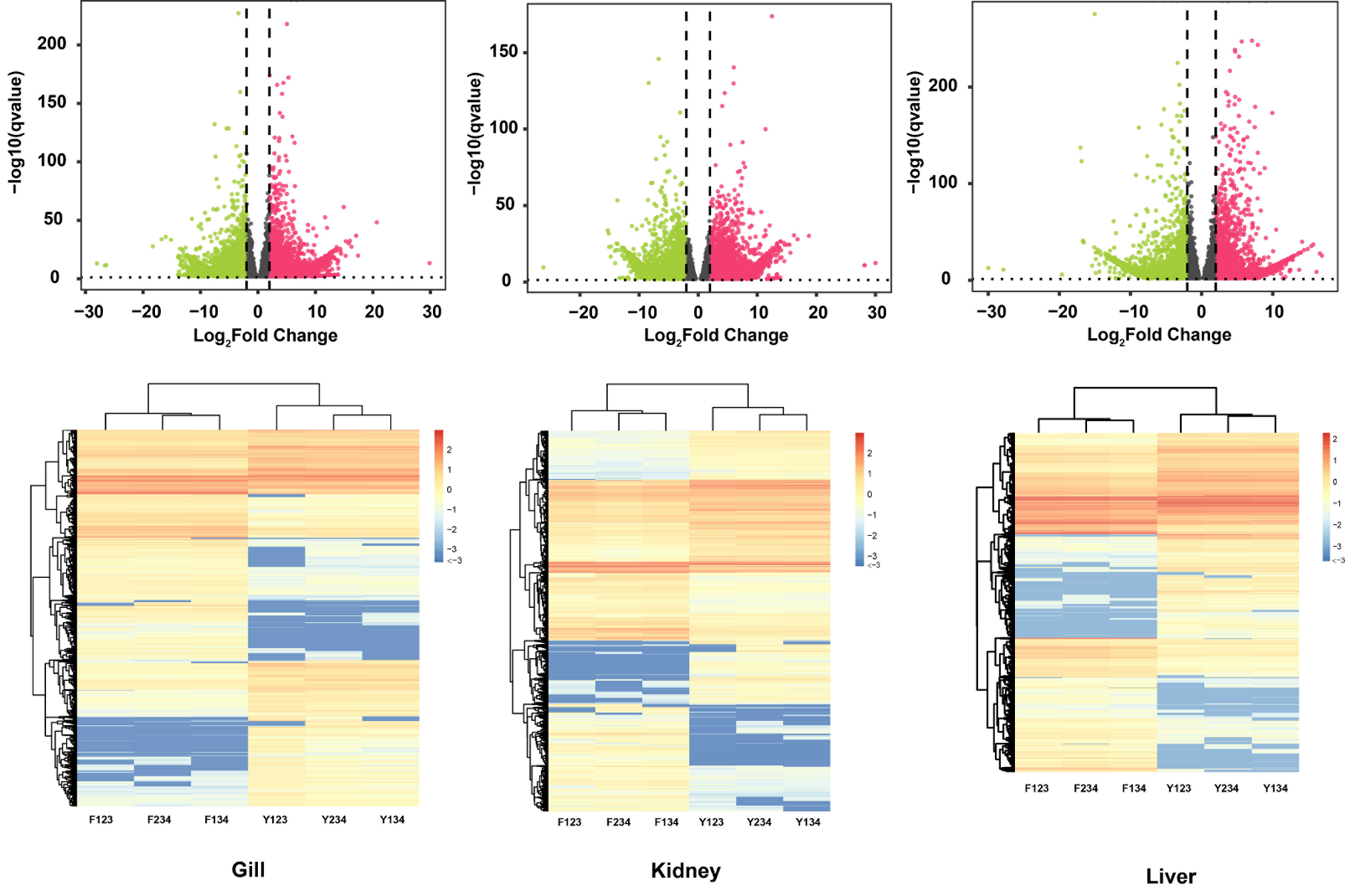
Fig S12. The volcanic map and the heatmap of differential expression analysis in the gill, liver and kidney of Amur Ide.

## Fig S13. The population genetic of the *L. waleckii* populations. (A) Maximum likelihood tree of the relationships between the 85 *L. waleckii* samples based on SNPs. Individuals from different populations are represented by different colours. (B) 3D plot visualizing the principal component analysis (PCA).


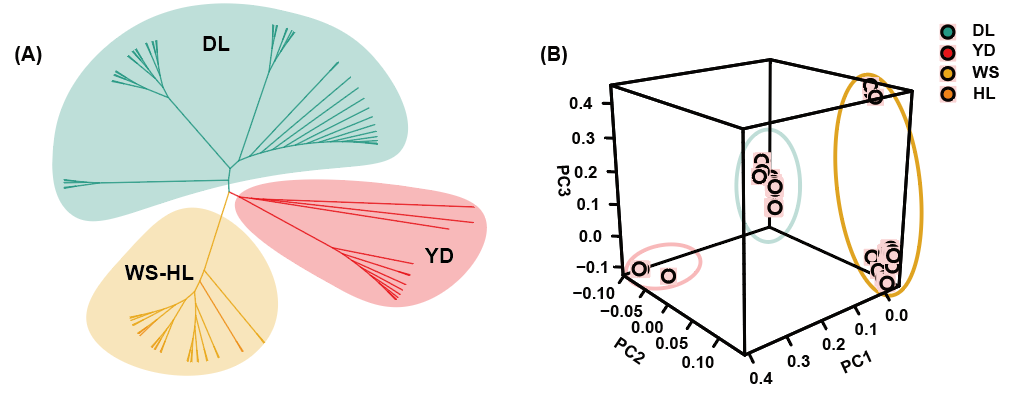


## Fig S14*.* (A) Distribution of monsoon in Northeast Asia and the location of Lake Dali Nur; (B) The schematic diagram of the drainage area of the ancient Paleolake and current Lake Dali Nur

**
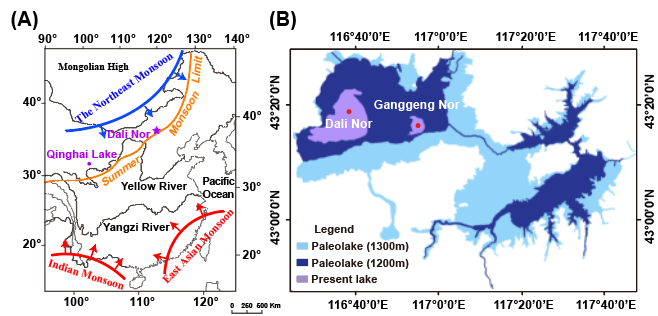
**

##
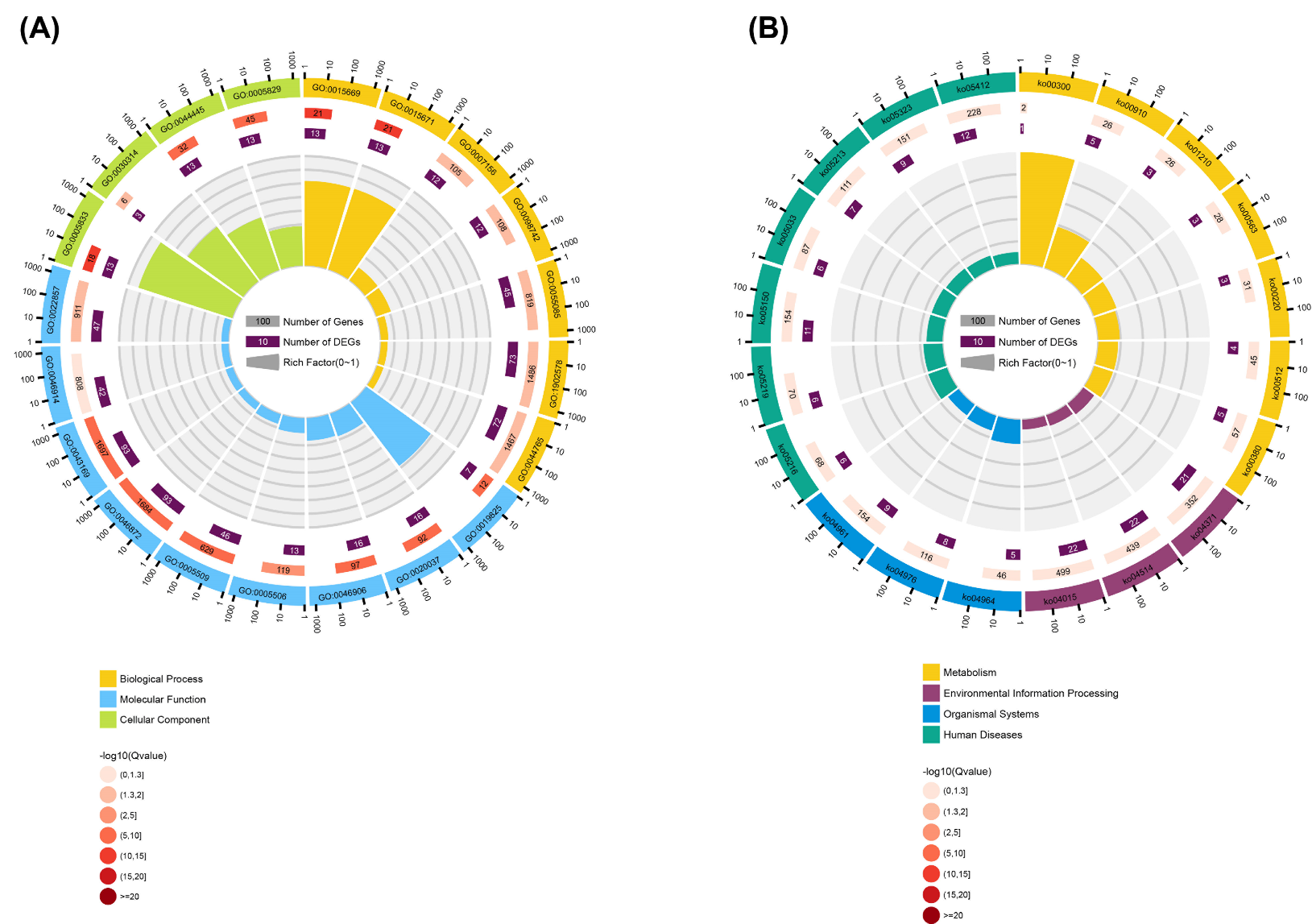
Fig S15. The enrichment of candidate selected genes.

**(A) The GO enrichment of candidate selected genes identified by Fst. (B) The GO enrichment of candidate selected genes is identified by π ratio.**

## Fig S16. The venn diagram of number of genes among DEGs and candidate selected genes.


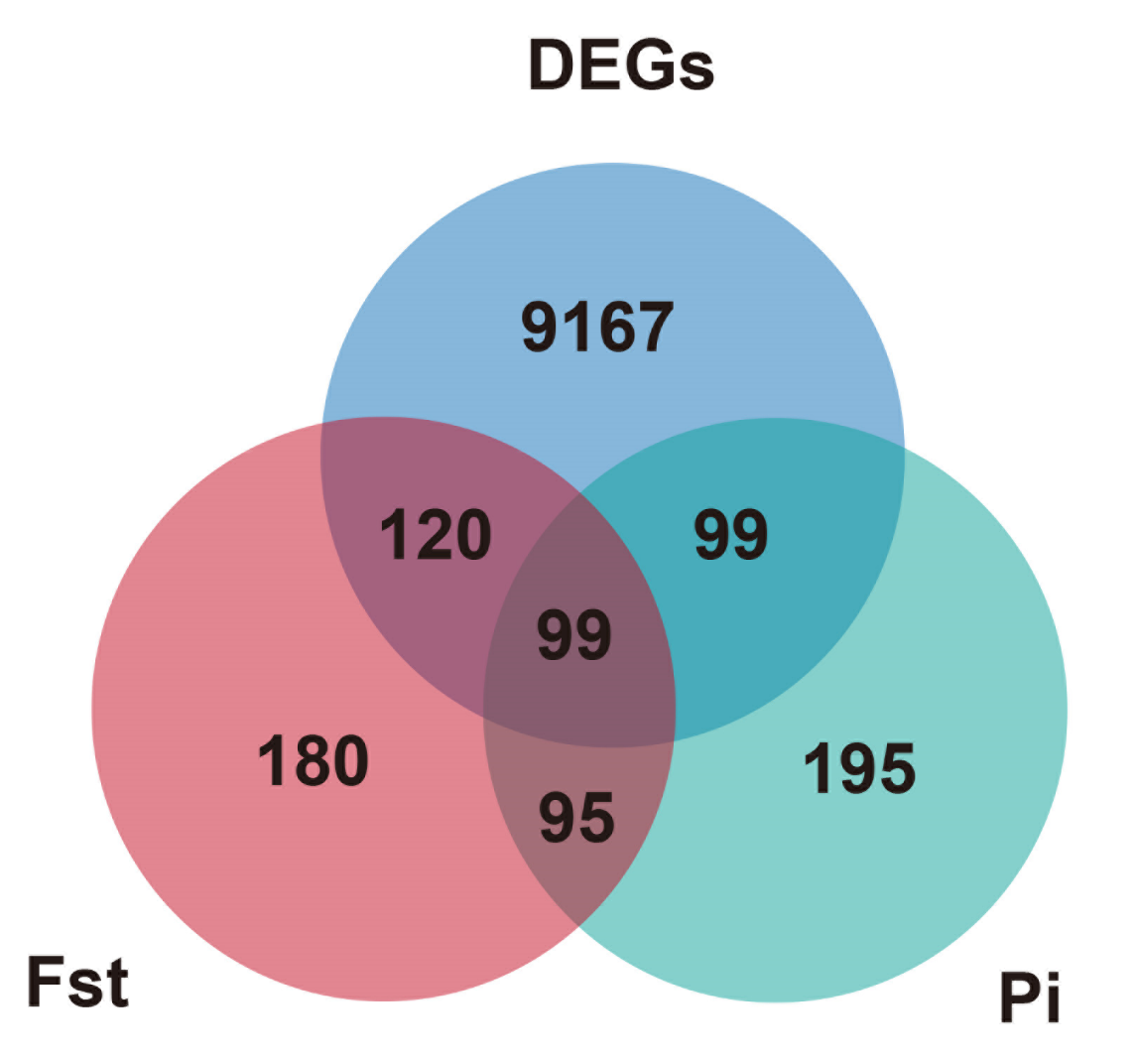


## Fig S17. The phylogenetic tree of CA genes between Amur Ide and releted species.

**
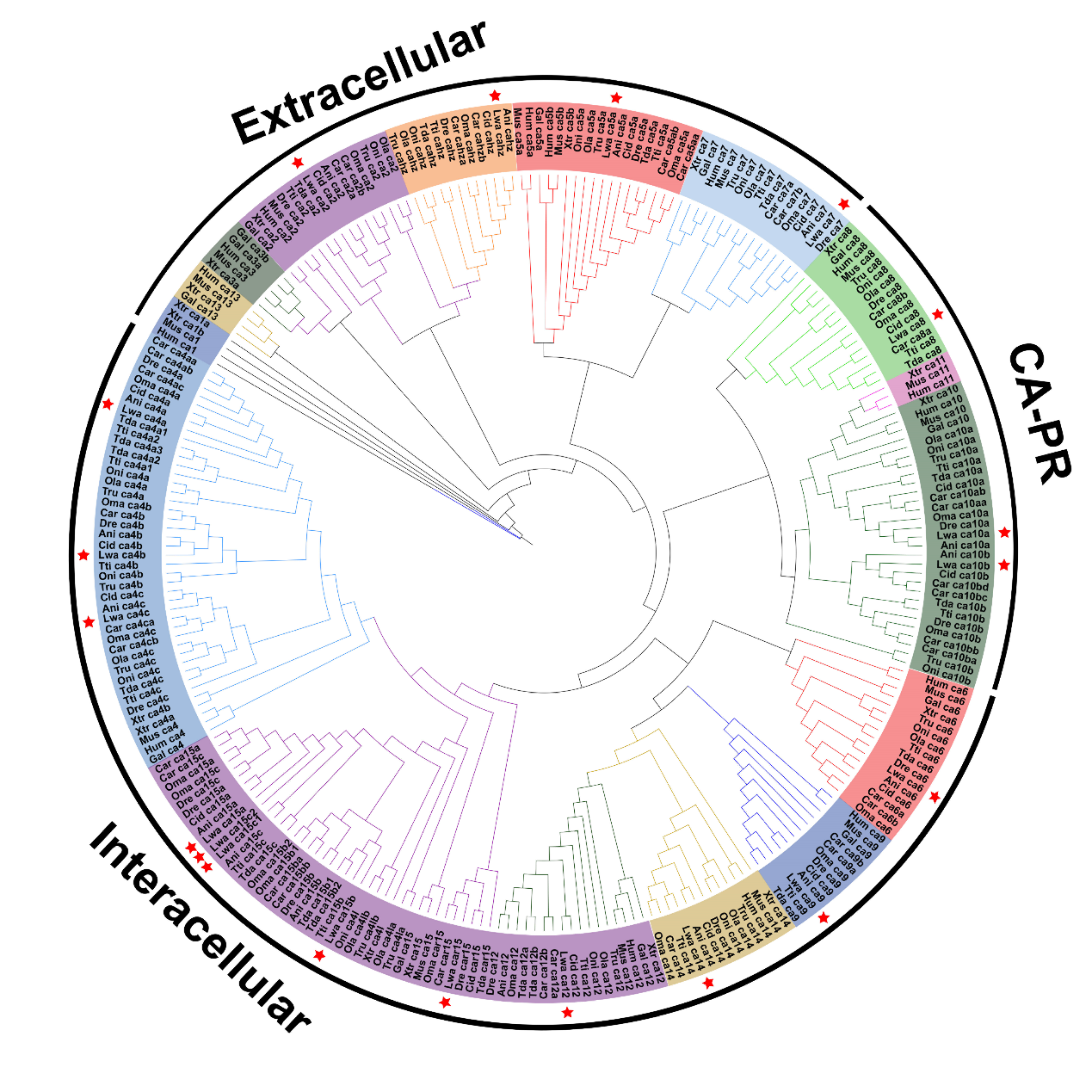
**19 CA genes of *L. waleckii* are marked by the red star. The abbreviation of species name is listed before gene name; Hum: *H. sapiens*, Mus: *M. musculus*, Gal: *G. gallus*, Xtr: *X. tropicalis*, Dre: *D. rerio*, Gac: *G. aculeatus*, Oni: *O. niloticus*, Tru: *T. rubripes*, Ola: *O. latipes*, Ani: *A. nigrocauda*, Car: *C. auratus*, Cid: *C. idella*, Oma: *O. macrolepis*, Tda: *T. dalaica*, and Tti: *T. tibetana***.**

## Fig S18. The motif of 19 CA genes in Amur Ide.

**
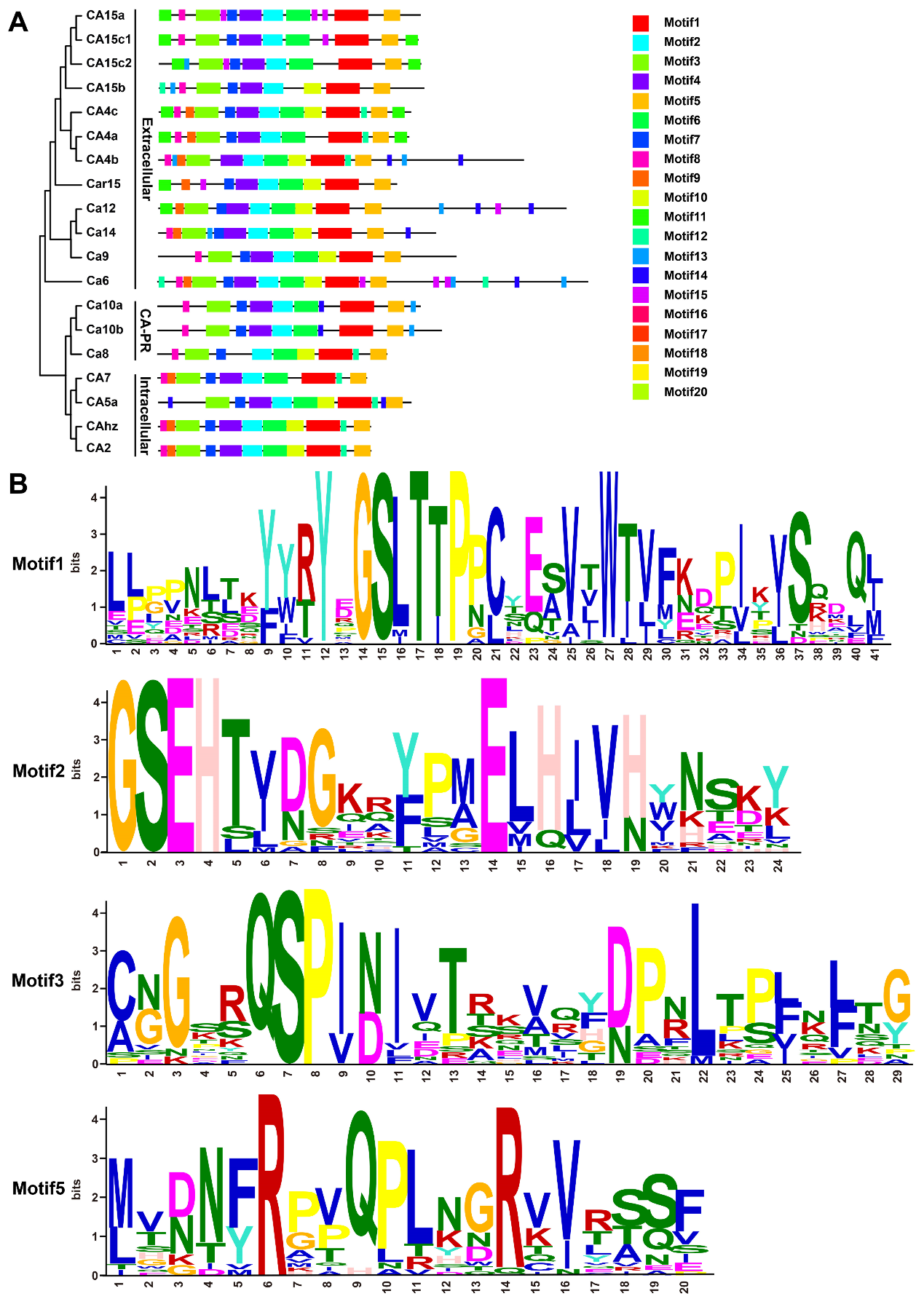
**

(A) The motif distributions of 19 CA genes in Amur Ide; (B) The shared 4 motifs of 19 CA genes in Amur Ide.

**Fig S19. The 3D structure of 3 copies of CA15 in the ALK *L. waleckii* population.**


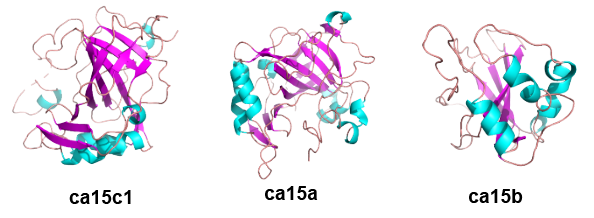


## Fig S20. The phylogenetic tree of RH glycoproteins between Amur Ide and releted species.

**
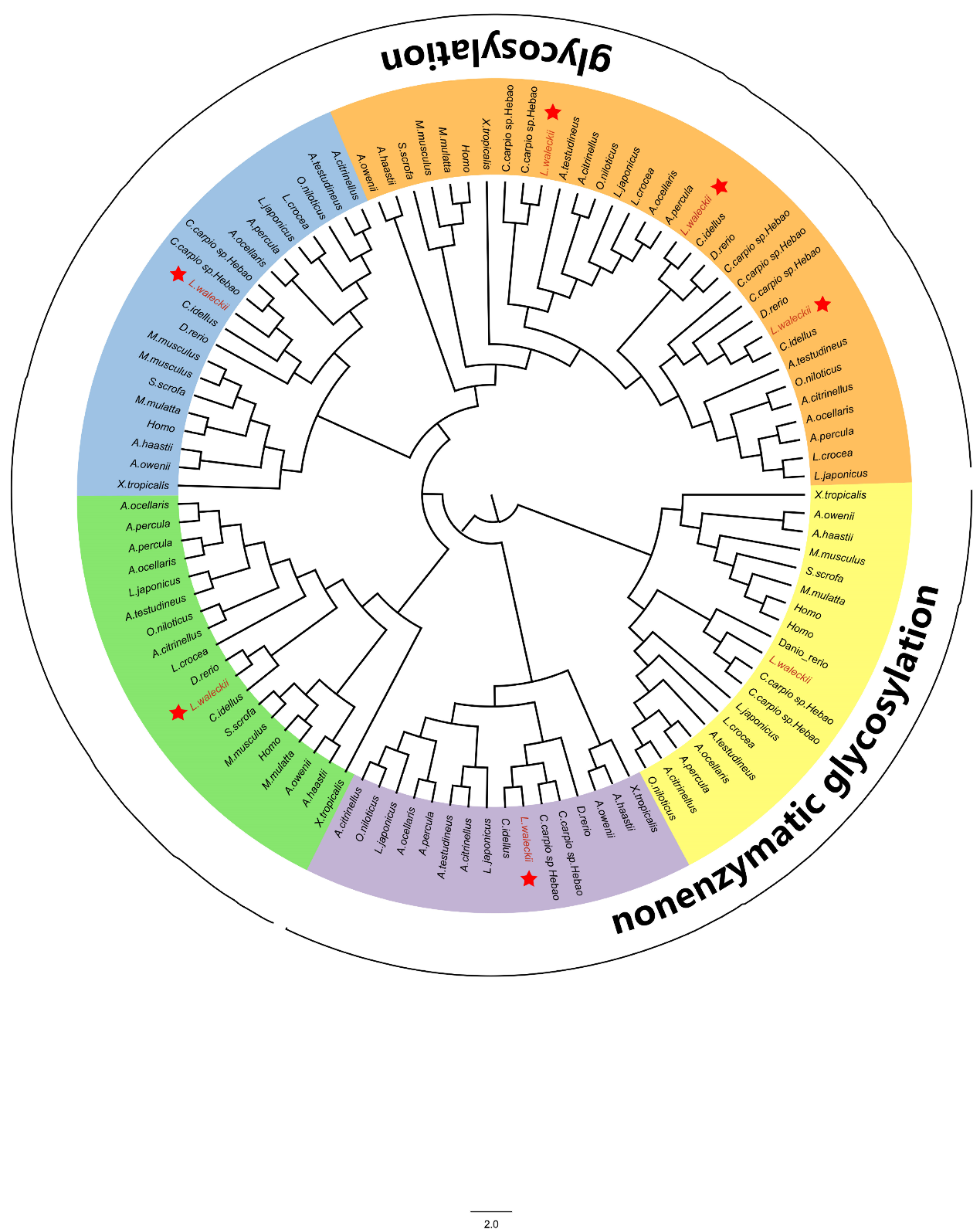
**

7 RH genes of *L. waleckii* are marked by the red star. Green: *rhag*; Blue: *rhbg*; Orange: *rhcg*; Purple: *rh50*; Yellow: *rhd*

## Fig S21. The motif of 7 RH genes in Amur Ide.

**
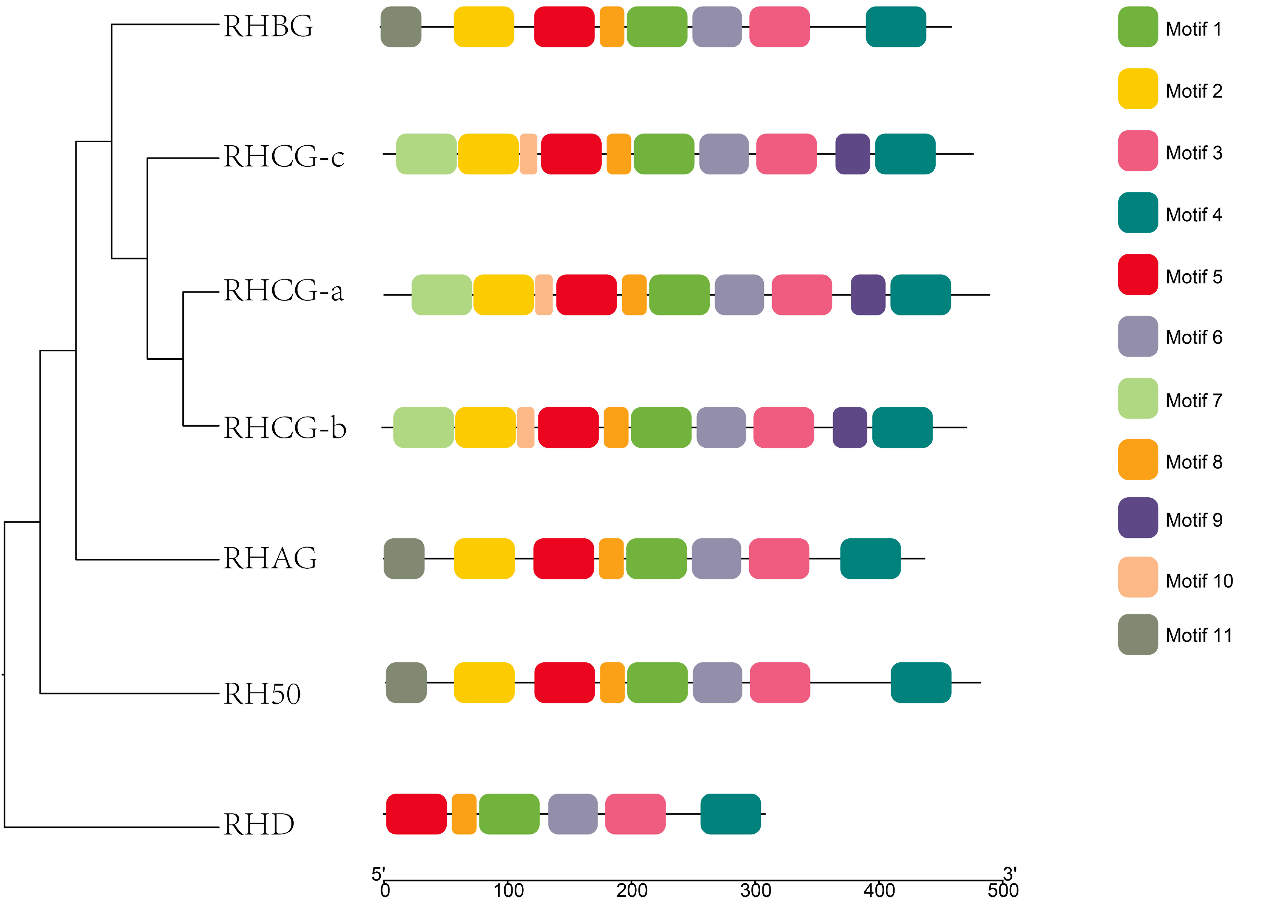
**

## Fig S22. The 3D structure of RHCG-a of ALK and FW *L. waleckii* population.

**
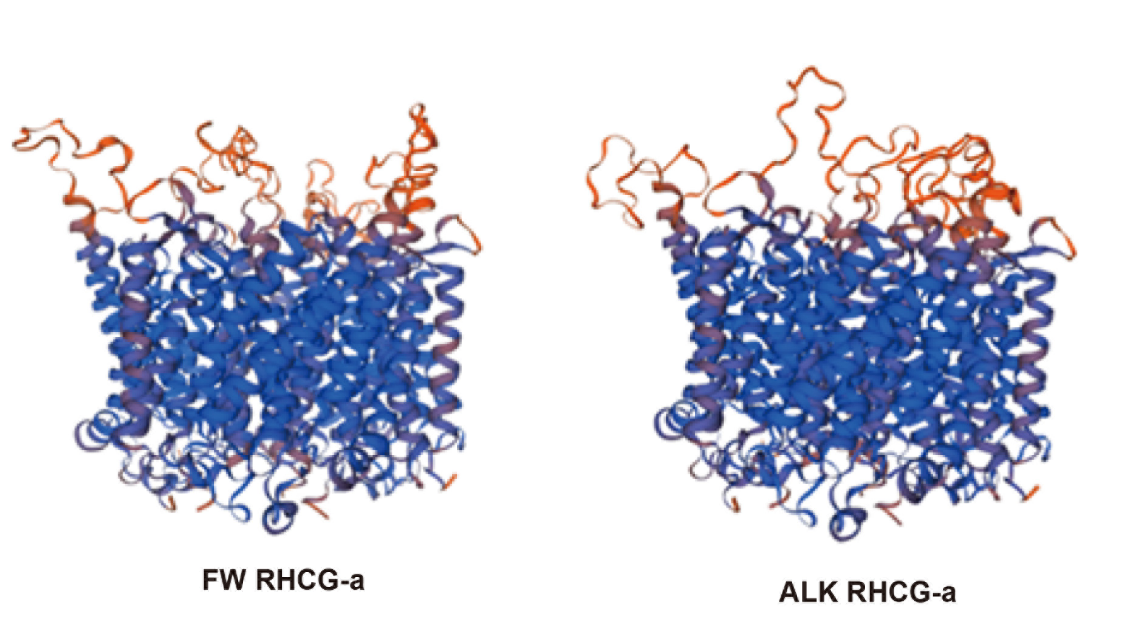
**

# Supplementary Table

**Table S1. The statistics of genome sequencing data.**

| Pair-end libraries | Insert size | Total data (G) | Read Length | Sequence coverage (X) |
| --- | --- | --- | --- | --- |
| Illumina reads | 350bp | 73 | 150 | 64.6 |
| PacBio reads | - | 62 | - | 54.87 |
| HiC | - | 102.93 | 150 | 92.35 |
| Total | - | 120.77 | - | 199.23 |

## Table S2. The statistics of 17 K-mer analysis of *L. waleckii*.

| Depth | number of kmer | Genome_size(M) | Revised Genome_size(M) | Heterozygous_rate(%) | Repeat_rate(%) |
| --- | --- | --- | --- | --- | --- |
| 43 | 49238269810 | 1145.08 | 1125.03 | 0.56 | 57.61 |

## Table S3. The statistics of PacBio Sequencing.

| Library | Read base (G) | Read number | Read length（Mean） | N50 |
| --- | --- | --- | --- | --- |
| PacBio | 73 | 2,030,960 | 35,943 | 62,499 |

## Table S4. The statistics of assembly of Amur Ide genome.

|  | Contig Length (bp) | Contig number |
| --- | --- | --- |
| Total | 1,103,966,172 | 6,407 |
| Max | 12,092,634 | - |
| Length>=2000 |  | 6,406 |
| N50 | 1,515,867 | 180 |
| N60 | 1,017,170 | 270 |
| N70 | 681,883 | 405 |
| N80 | 336,993 | 630 |
| N90 | 95,719 | 1,246 |
| GC content | 0.3877 |  |

## Table S5. The statistics of Repeat elements annotation of Amur Ide genome.

| Type | Repeat Size (bp) | % in genome |
| --- | --- | --- |
| Trf | 69,759,993 | 6.32 |
| Repeatmasker | 524,034,603 | 47.47 |
| Proteinmask | 82,731,199 | 7.49 |
| Total | 551,058,658 | 49.92 |

## Table S6. Detailed classification of repeat sequences in Amur Ide genome.

(Included in a separated excel file)

## Table S7. The statistics of gene structure annotation of Amur Ide genome.

(Included in a separated excel file)

## Table S8. The statistics of gene function annotation of Amur Ide genome.

|  | Number | Percent (%) |
| --- | --- | --- |
| Total | 27633 | - |
| Swissprot | 22629 | 81.9 |
| Nr | 26579 | 96.2 |
| KEGG | 23536 | 85.2 |
| InterPro | 24392 | 88.3 |
| GO | 16660 | 60.3 |
| Pfam | 21205 | 76.7 |
| Annotated | 26613 | 96.3 |
| Unannotated | 1020 | 3.7 |

## Table S9. The statistics of chromosome assembly of Amur Ide.

| chromosome | Length |
| --- | --- |
| chr3 | 71368983 |
| chr1 | 63702483 |
| chr7 | 62271240 |
| chr5 | 49313439 |
| chr4 | 45682451 |
| chr2 | 42701760 |
| chr9 | 41820828 |
| chr15 | 41008407 |
| chr19 | 40980234 |
| chr22 | 40598971 |
| chr18 | 40463193 |
| chr6 | 39639001 |
| chr14 | 37199493 |
| chr16 | 37024483 |
| chr21 | 36678393 |
| chr20 | 35751175 |
| chr12 | 35278759 |
| chr17 | 35180327 |
| chr8 | 34866635 |
| chr23 | 34564867 |
| chr13 | 34473392 |
| chr24 | 32144632 |
| chr11 | 30489377 |
| chr25 | 28728858 |
| chr10 | 28415676 |
| sum | 1020347057 |
| N50 | 39639001 |
| Total | 1105256174 |

## Table S10. The BUSCO survey of Amur Ide.

| Species | BUSCO notation assessment results |
| --- | --- |
| *L.waleckii* | C:96.4%[S:93.6%,D:2.8%],F:2.4%,M:1.2%,n:4,584 |

## Table S11. The mapping statistics of Illumina sequencing reads to reference Amur Ide genome.

|  |  | Percentage |
| --- | --- | --- |
| Reads | Mapping rate (%) | 99.05 |
| Genome | Average sequencing depth | 48.66 |
|  | Coverage (%) | 93.65 |
|  | Coverage at least 4X (%) | 92.19 |
|  | Coverage at least 10X (%) | 90.82 |

## Table S12. The statistics of SNPs in the genome sequencing individual.

|  | Number | Percentage |
| --- | --- | --- |
| All SNP | 2,707,134 | 0.2756 |
| Heterozygosis SNP | 2,662,101 | 0.271 |
| Homology SNP | 45,033 | 0.0046 |

## Table S13. The statistics of orthologues gene families between Amur Ide and related species.

(Included in a separated excel file)

## Table S14. The statistics of detailed classification of transposable element sequences between Amur Ide and related species*.*

(Included in a separated excel file)

## Table S15. The statistics of expansion and contraction gene families between *L. waleckii* and related species.

| Species | Expansion | Contractions | Other | Total |
| --- | --- | --- | --- | --- |
| Tti | 1123 | 2705 | 21022 | 24850 |
| Tda | 1736 | 1372 | 21742 | 24850 |
| Dre | 4421 | 6930 | 13499 | 24850 |
| Lro | 3598 | 7706 | 13546 | 24850 |
| Oma | 1330 | 2732 | 20788 | 24850 |
| Lwa | 1751 | 5202 | 17897 | 24850 |
| Cid | 2733 | 6227 | 19384 | 24850 |
| Asg | 1395 | 8495 | 14960 | 24850 |
| Ani | 2270 | 3622 | 18958 | 24850 |
| Ola | 1965 | 10237 | 12648 | 24850 |

## Table S16. The GO enrichment of expansion gene families in Amur Ide*.*

(Included in a separated excel file)

## Table S17. The GO enrichment of contraction gene families in Amur Ide.

(Included in a separated excel file)

## Table S18. The gene numbers of GGT gene family between Amur Ide and related species.

|  | Agr | Ani | Cid | Lwa | Oma | Dre | Tda | Tti | Ola |
| --- | --- | --- | --- | --- | --- | --- | --- | --- | --- |
| *ggt1* | 2 | 1 | 2 | 2 | 1 | 2 | 2 | 2 | 0 |
| *ggt1l* | 1 | 1 | 4 | 8 | 2 | 2 | 2 | 1 | 0 |
| *ggt5* | 2 | 1 | 2 | 2 | 1 | 2 | 2 | 2 | 0 |
| *ggt6* | 1 | 1 | 1 | 1 | 1 | 0 | 1 | 1 | 0 |
| *ggt7* | 0 | 1 | 1 | 1 | 1 | 1 | 1 | 1 | 3 |
| sum | 6 | 5 | 10 | 14 | 6 | 7 | 8 | 7 | 3 |

## Table S19. The rapid evolution genes and function annotation in Amur Ide.

(Included in a separated excel file)

## Table S20. The GO enrichment of rapid evolution genes in Amur Ide.

(Included in a separated excel file)

## Table S21. The positively selected genes and function annotation in Amur Ide.

(Included in a separated excel file)

## Table S22. The GO enrichment of positively selected genes in Amur Ide.

(Included in a separated excel file)

## Table S23. The statistics of ReviGO analysis of rapid evolution genes in Amur Ide.

(Included in a separated excel file)

## Table S24. The statistics of ReviGO analysis of positively selected genes in Amur Ide.

(Included in a separated excel file)

## Table S25. The statistics of RNA sequencing between ALK and FW Amur Ide population.

| sample | Tissue | raw_reads | clean_reads | clean_bases | error_rate | Q20 | Q30 | GC_pct |
| --- | --- | --- | --- | --- | --- | --- | --- | --- |
| Y123 | Gill | 23839355 | 23341780 | 7G | 0.03 | 97.84 | 93.83 | 40.86 |
| Y234 | Gill | 26461363 | 25944003 | 7.78G | 0.03 | 97.81 | 93.81 | 44.38 |
| Y134 | Gill | 24263567 | 23811428 | 7.14G | 0.03 | 97.88 | 93.99 | 43.75 |
| Y123 | Kidney | 23805989 | 23450813 | 7.04G | 0.02 | 98.06 | 94.39 | 45.29 |
| Y234 | Kidney | 22718146 | 22295627 | 6.69G | 0.03 | 97.96 | 94.11 | 45.58 |
| Y134 | Kidney | 23641964 | 23269140 | 6.98G | 0.02 | 98.23 | 94.69 | 45.17 |
| Y123 | Liver | 23113754 | 22706268 | 6.81G | 0.02 | 98.25 | 94.81 | 46.7 |
| Y234 | Liver | 24854195 | 24405169 | 7.32G | 0.02 | 98.49 | 95.28 | 46.9 |
| Y134 | Liver | 25939636 | 25413351 | 7.62G | 0.02 | 98.41 | 95.17 | 46.86 |
| F123 | Gill | 23851172 | 23304759 | 6.99G | 0.02 | 98.3 | 94.94 | 45.7 |
| F234 | Gill | 23483358 | 22992644 | 6.9G | 0.02 | 98.13 | 94.54 | 45.82 |
| F134 | Gill | 27551393 | 27010135 | 8.1G | 0.02 | 98.15 | 94.53 | 46.17 |
| F123 | Kidney | 23977208 | 23258985 | 6.98G | 0.02 | 98.27 | 95.03 | 46.75 |
| F234 | Kidney | 23263563 | 22797508 | 6.84G | 0.03 | 97.95 | 94.24 | 46.49 |
| F134 | Kidney | 23284498 | 22866900 | 6.86G | 0.02 | 98.35 | 95.07 | 46.55 |
| F123 | Liver | 23754957 | 23306632 | 6.99G | 0.03 | 97.69 | 93.37 | 46.63 |
| F234 | Liver | 23416841 | 23029748 | 6.91G | 0.02 | 98.49 | 95.22 | 46.79 |
| F134 | Liver | 27891699 | 27203087 | 8.16G | 0.02 | 98.49 | 95.25 | 47.14 |
| sum | - | 439112658 | 430407977 | 129.11G | - | - | - | - |

## Table S26. The statistics of gene expression in gill between ALK and FW Amur Ide population.

(Included in a separated excel file)

## Table S27. The up-regulated gene list of ALK Amur Ide population in gill.

(Included in a separated excel file)

## Table S28. The down-regulated gene list of ALK Amur Ide population in gill.

(Included in a separated excel file)

## Table S29. The statistics of gene expression in kidneys between ALK and FW Amur Ide population.

(Included in a separated excel file)

## Table S30. The up-regulated gene list of ALK Amur Ide population in the kidney.

(Included in a separated excel file)

## Table S31. The down-regulated gene list of ALK Amur Ide population in the kidney.

(Included in a separated excel file)

## Table S32. The statistics of gene expression in liver between ALK and FW Amur Ide population.

(Included in a separated excel file)

## Table S33. The up-regulated gene list of ALK Amur Ide population in the liver.

(Included in a separated excel file)

## Table S34. The down-regulated gene list of ALK Amur Ide population in the liver.

(Included in a separated excel file)

## Table S35. The GO enrichment of up-regulated gene of ALK Amur Ide population in gill.

(Included in a separated excel file)

## Table S36. The GO enrichment of down-regulated gene of ALK Amur Ide population in gill.

(Included in a separated excel file)

## Table S37. The GO enrichment of up-regulated gene of ALK Amur Ide population in kidney.

(Included in a separated excel file)

## Table S38. The GO enrichment of down-regulated gene of ALK Amur Ide population in kidney.

(Included in a separated excel file)

## Table S39. The GO enrichment of up-regulated gene of ALK Amur Ide population in liver.

(Included in a separated excel file)

## Table S40. The GO enrichment of down-regulated gene of ALK Amur Ide population in liver.

(Included in a separated excel file)

## Table S41. The rapid evolution genes with differential expression in ALK population.

(Included in a separated excel file)

## Table S42. The positively selected genes with differential expression in ALK population.

(Included in a separated excel file)

Note：The separated excel file could be found via

## Table S43. The statistics of Genome Resequencing between ALK and FW Amur Ide population.

(Included in a separated excel file)

## Table S44. The statistics of SNPs between 4 Amur Ide population.

| Category | DL | WS | YD | HL | ALL |
| --- | --- | --- | --- | --- | --- |
| SNPs | 3513387 | 3922726 | 3122707 | 2855556 | 6206224 |
| Intergenic SNPs | 2295142 | 2539536 | 2013242 | 1872427 | 4033100 |
| SNPs in Gene | 1218245 | 1383190 | 1109465 | 983129 | 2173124 |
| Intronic SNPs | 1061562 | 1220455 | 977574 | 869144 | 1929389 |
| Exonic SNPs | 156683 | 162735 | 131891 | 113985 | 243735 |
| SNPs in 3’-UTR | 24086 | 27000 | 21665 | 16668 | 41816 |
| SNPs in 5’-UTR | 3090 | 2830 | 2285 | 1493 | 5756 |
| SNPs in CDS | 129507 | 132905 | 107941 | 95824 | 196163 |
| No. INDELs | 110316 | 274921 | 368693 | 416994 | 845427 |

## Table S45. The statistics of Fst and π ratio scanning in 25 chromosomes between ALK and FW Amur Ide population.

| Chromosome | median of π | | median of ρ | |
| --- | --- | --- | --- | --- |
|  | ALK | FW | ALK | FW |
| 1 | 0.000981186 | 0.00102222 | 109.5369838 | 142.8797446 |
| 2 | 0.00113694 | 0.00124035 | 110.456985 | 163.4349425 |
| 3 | 0.00106511 | 0.001086 | 130.6563018 | 147.8673014 |
| 4 | 0.00107276 | 0.0012121 | 110.7087671 | 158.3597406 |
| 5 | 0.00112776 | 0.00118218 | 109.4068606 | 157.323508 |
| 6 | 0.00101668 | 0.00112334 | 92.76513626 | 160.3161602 |
| 7 | 0.00105044 | 0.00109264 | 109.8517279 | 153.8119188 |
| 8 | 0.00119754 | 0.0012819 | 113.9412327 | 169.3761975 |
| 9 | 0.00110195 | 0.00118365 | 102.2559117 | 158.5018678 |
| 10 | 0.00120777 | 0.00136961 | 105.4763692 | 177.1545242 |
| 11 | 0.00104441 | 0.00120533 | 90.83679528 | 172.8768996 |
| 12 | 0.000965553 | 0.00112858 | 82.60421771 | 167.1634125 |
| 13 | 0.0012115 | 0.00125448 | 105.1378618 | 173.3711374 |
| 14 | 0.0011941 | 0.00121113 | 121.3783641 | 166.766422 |
| 15 | 0.00110581 | 0.0011551 | 116.7737874 | 154.4111877 |
| 16 | 0.00112454 | 0.00119826 | 103.3940643 | 166.9325362 |
| 17 | 0.00115974 | 0.00122642 | 101.1232752 | 168.8686508 |
| 18 | 0.00110132 | 0.00119132 | 101.1886091 | 160.6208974 |
| 19 | 0.00114627 | 0.00114823 | 119.879374 | 161.8801581 |
| 20 | 0.00100791 | 0.00116224 | 94.20244469 | 166.4233426 |
| 21 | 0.00110343 | 0.0011886 | 102.7857523 | 164.0646555 |
| 22 | 0.00108238 | 0.00113934 | 133.3337419 | 157.3361366 |
| 23 | 0.000993889 | 0.0011473 | 84.33045281 | 164.3682196 |
| 24 | 0.00104489 | 0.00117884 | 99.18769828 | 164.6084054 |
| 25 | 0.00119587 | 0.00124743 | 117.9801652 | 167.1187866 |
| Average | 0.00120804 | 0.00128831 | 121.513 | 150.838 |
| P-value of T-test | 1.10E-04 | | 1.01E-23 | |

## Table S46. The candidate selected regions that were identified by Fst in ALK Amur Ide population.

(Included in a separated excel file)

## Table S47. The candidate genes that were identified by Fst in ALK Amur Ide population.

(Included in a separated excel file)

## Table S48. The candidate selected regions that identified by π ratio in ALK Amur Ide population.

(Included in a separated excel file)

## Table S49. The candidate genes that were identified by π ratio in ALK Amur Ide population.

(Included in a separated excel file)

## Table S50. The GO enrichment of candidate selected genes in ALK Amur Ide population.

(Included in a separated excel file)

## Table S51. The KEGG enrichment of candidate selected genes in ALK Amur Ide population.

(Included in a separated excel file)

## Table S52. The copy numbers of CA genes between Amur Ide and related species.

## Table S53. The accession number of CA genes in Cyprinidae fish.

(Included in a separated excel file)

## Table S54. The statistics of 19 CA genes in ALK Amur Ide population.

(Included in a separated excel file)

## Table S55. The statistics of highly differentiated sites in CA genes between ALK and FW Amur Ide population.

(Included in a separated excel file)

## Table S56. The accession number of RH genes in Cyprinidae fish.

(Included in a separated excel file)

## Table S57. The statistics of 7 RH genes in ALK Amur Ide population.

(Included in a separated excel file)

## Table S58. The statistics of highly differentiated sites in *rhcga* between ALK and FW Amur Ide population.

(Included in a separated excel file)

**Note: The separated excel file was uploaded on figshare database, which can be acess via https://doi.org/10.6084/m9.figshare.22294135.v1**

**Reference**

1. Zhou, Z.X. *et al.* The sequence and de novo assembly of Takifugu bimaculatus genome using PacBio and Hi-C technologies. *Scientific Data* **6**(2019).

2. Cox, M.P., Peterson, D.A. & Biggs, P.J. SolexaQA: At-a-glance quality assessment of Illumina second-generation sequencing data. *BMC Bioinformatics* **11**, 485 (2010).

3. Xu, P. *et al.* Genome sequence and genetic diversity of the common carp, Cyprinus carpio. *Nat Genet* **46**, 1212-9 (2014).

4. Koren, S. *et al.* Canu: scalable and accurate long-read assembly via adaptive k-mer weighting and repeat separation. *Genome Research* **27**, 722-736 (2017).

5. Myers, E.W. The fragment assembly string graph. *Bioinformatics* **21 Suppl 2**, ii79-85 (2005).

6. Walker, B.J. *et al.* Pilon: an integrated tool for comprehensive microbial variant detection and genome assembly improvement. *PLoS One* **9**, e112963 (2014).

7. Li, H. & Durbin, R. Fast and accurate short read alignment with Burrows-Wheeler transform. *Bioinformatics* **25**, 1754-1760 (2009).

8. Abrusan, G., Grundmann, N., DeMester, L. & Makalowski, W. TEclass--a tool for automated classification of unknown eukaryotic transposable elements. *Bioinformatics* **25**, 1329-30 (2009).

9. Schemberger, M.O. *et al.* DNA transposon invasion and microsatellite accumulation guide W chromosome differentiation in a Neotropical fish genome. *Chromosoma* **128**, 547-560 (2019).

10. Benson, G. Tandem repeats finder: a program to analyze DNA sequences. *Nucleic Acids Research* **27**, 573-580 (1999).

11. Xu, P. *et al.* The allotetraploid origin and asymmetrical genome evolution of the common carp Cyprinus carpio. *Nat Commun* **10**, 4625 (2019).

12. Howe, K. *et al.* The zebrafish reference genome sequence and its relationship to the human genome (vol 496, pg 498, 2013). *Nature* **505**, 248-248 (2014).

13. Wang, Y.P. *et al.* The draft genome of the grass carp (Ctenopharyngodon idellus) provides insights into its evolution and vegetarian adaptation. *Nature Genetics* **47**, 625-631 (2015).

14. Yang, J.X. *et al.* The Sinocyclocheilus cavefish genome provides insights into cave adaptation. *Bmc Biology* **14**(2016).

15. Xu, J. *et al.* Genomic Basis of Adaptive Evolution: The Survival of Amur Ide (Leuciscus waleckii) in an Extremely Alkaline Environment. *Molecular Biology and Evolution* **34**, 145-159 (2017).

16. Kent, W.J. BLAT - The BLAST-like alignment tool. *Genome Research* **12**, 656-664 (2002).

17. Birney, E., Clamp, M. & Durbin, R. GeneWise and Genomewise. *Genome Res* **14**, 988-95 (2004).

18. Stanke, M. & Morgenstern, B. AUGUSTUS: a web server for gene prediction in eukaryotes that allows user-defined constraints. *Nucleic Acids Research* **33**, W465-W467 (2005).

19. Majoros, W.H., Pertea, M. & Salzberg, S.L. TigrScan and GlimmerHMM: two open source ab initio eukaryotic gene-finders. *Bioinformatics* **20**, 2878-9 (2004).

20. Korf, I. Gene finding in novel genomes. *BMC Bioinformatics* **5**, 59 (2004).

21. Parra, G., Blanco, E. & Guigo, R. GeneID in Drosophila. *Genome Res* **10**, 511-5 (2000).

22. Burge, C. & Karlin, S. Prediction of complete gene structures in human genomic DNA. *J Mol Biol* **268**, 78-94 (1997).

23. Trapnell, C., Pachter, L. & Salzberg, S.L. TopHat: discovering splice junctions with RNA-Seq. *Bioinformatics* **25**, 1105-11 (2009).

24. Trapnell, C. *et al.* Transcript assembly and quantification by RNA-Seq reveals unannotated transcripts and isoform switching during cell differentiation. *Nat Biotechnol* **28**, 511-5 (2010).

25. Haas, B.J. *et al.* Automated eukaryotic gene structure annotation using EVidenceModeler and the program to assemble spliced alignments. *Genome Biology* **9**(2008).

26. Haas, B.J. *et al.* Improving the Arabidopsis genome annotation using maximal transcript alignment assemblies. *Nucleic Acids Research* **31**, 5654-5666 (2003).

27. Jones, P. *et al.* InterProScan 5: genome-scale protein function classification. *Bioinformatics* **30**, 1236-40 (2014).

28. Moriya, Y., Itoh, M., Okuda, S., Yoshizawa, A.C. & Kanehisa, M. KAAS: an automatic genome annotation and pathway reconstruction server. *Nucleic Acids Res* **35**, W182-5 (2007).

29. Kim, D., Langmead, B. & Salzberg, S.L. HISAT: a fast spliced aligner with low memory requirements. *Nat Methods* **12**, 357-60 (2015).

30. Pertea, M., Kim, D., Pertea, G.M., Leek, J.T. & Salzberg, S.L. Transcript-level expression analysis of RNA-seq experiments with HISAT, StringTie and Ballgown. *Nat Protoc* **11**, 1650-67 (2016).

31. Wang, X. *et al.* Genomic basis of evolutionary adaptation in a warm-blooded fish. *The Innovation* **3**(2022).

32. Zhou, Z. *et al.* The sequence and de novo assembly of Takifugu bimaculatus genome using PacBio and Hi-C technologies. *Sci Data* **6**, 187 (2019).

33. Chen, L. *et al.* Chromosome-level genome of Poropuntius huangchuchieni provides a diploid progenitor-like reference genome for the allotetraploid Cyprinus carpio. *Mol Ecol Resour* **21**, 1658-1669 (2021).
